# Supplementary material for: The α-synuclein PET tracer [18F] ACI-12589 distinguishes multiple system atrophy from other neurodegenerative diseases
Source: Nat Commun. 2023 Oct 27;14:6750. doi: 10.1038/s41467-023-42305-3 (PMC10611796; doi:10.1038/s41467-023-42305-3)
Supplement: Supplementary file 1 — Supplementary Information [file 41467_2023_42305_MOESM1_ESM.pdf]

Supplementary information

The α-synuclein PET tracer [18F] ACI-12589 distinguishes multiple system atrophy from other neurodegenerative diseases

Index Page

Supplementary methods 2-7

Preparation of human brain homogenates from an AD donor 2

Preparation of human brain homogenates from a healthy control donor 2

Radiobinding assay using [^{3}H]ACI-12589 for determination of the dissociation constant (K_d) on AD brain homogenates 3

Radiobinding assay using [^{3}H]L-deprenyl for determination of the inhibition constant (K_i) on brain homogenates containing MAO-B 3

Kinetic analysis *4*

Radiosynthesis of [^{18}F]ACI-12589 5

Quality control – HPLC and TLC 6

Blood sampling and metabolite analysis 6-7

Suppl. Figure 1 Structure of ACI-12589 and precursor 8

Suppl. Figure 2 Autoradiography and saturation binding experiments. 9-10

Suppl. Figure 3 High resolution images from Figure 1c. 11

Suppl. Figure 4 AlphaLisa measurements of Aβ and tau from Fig 2a. 12

Suppl. Figure 5 Autoradiography and IHC in PSP patients 13

Suppl. Figure 6 TDP-43 autoradiography. 14

Suppl. Figure 7 Aβ and Tau staining of AD donor in Fig. 2c 15

Suppl. Table 1 Evaluation of K_d and Bmax in MSA and PD 16

Suppl. Table 2 Evaluation of K_d and Bmax of [^{3}H]ACI-12589 across different donors by micro-radiobinding 17

Suppl. Figure 8 *In vitro* off-target binding assays 18-20

Suppl. Figure 9 Time activity curves and parent fractions. 21

Suppl. Figure 10 Reference region data and kinetic modelling 22

Suppl. Figure 11 Images of the basal ganglia 23

Suppl. Figure 12 Images of the cerebellar white matter 24

Suppl. Figure 13 Supplementary ROIs 25-26

Suppl. Figure 14 Images of SNCA duplication carriers 27

Suppl. Figure 15 [^{18}F]ACI-12589 Correlations to [^{18}F]RO948 and [^{18}F]Flutemetamol 28-29

Suppl. Figure 16 Definition of the middle cerebellar peduncle ROI 30

Suppl. Table 3 Parameters used for analytical HPLC method 31

Suppl. Figure 17 Supplementary HPLC - parent fraction analysis 32

Suppl. Table 4 Human brain tissues for *in vitro* experiments 33

Suppl. Table 5 Neuropathology reports 34-35

Suppl. references 36

Supplementary methods

Preparation of human brain homogenates from an AD donor

The procedure used was adapted from Bagchi et al., 2013,[1] describing extraction of an insoluble fraction containing protein aggregates from human brain tissue for *in vitro* binding and competition studies. Frozen brain tissue samples were purchased from Tissue Solutions.

Frozen tissue from the frontal cortex brain region of an AD donor with confirmed burden of Tau and β-Amyloid– aggregates was used. The tissue was homogenized in high salt buffer (50mM Tris-HCl pH 7.5, 0.75M NaCl, 5mM EDTA) supplemented with protease inhibitors (Complete; Roche 11697498001) at 4°C using a glass Dounce homogenizer. The homogenate was transferred into polycarbonate centrifuge bottles (16 x 76mm; Beckman 355603) and centrifuged at 100,000 x g (38,000 RPM) in an ultracentrifuge (Beckman, XL100K) for 60 minutes at 4°C using a pre-cooled 70.1 rotor (Beckman, 342184). Pellets were resuspended in high salt buffer supplemented with 1% Triton X-100 and homogenized at 4°C. The homogenates were centrifuged again at 100,000 x g (38,000 RPM, 70.1 Ti rotor) for 60 minutes at 4°C. Pellets were resuspended in high salt buffer supplemented with 1% Triton X-100 and 1M sucrose at 4°C. The homogenates were centrifuged at 100,000 x g (38,000 RPM, 70.1 Ti rotor) for 60 minutes at 4°C. The resulting pellets containing β-Amyloid and Tau aggregates were resuspended in PBS, aliquoted and stored at -80°C until use.

Preparation of human brain homogenates from a healthy control donor

Frozen human tissue sample from the thalamus brain region, previously reported to display increased levels of MAO-B expression (Tong et al., 2013)[2], was purchased from Tissue Solutions. Approximately 2g of frozen tissue from the thalamus brain region of a healthy control donor was used. The tissue was homogenized in high salt buffer (25mM Tris-HCl pH 7.5, 0.15M NaCl, 1mM EDTA, 1mM EGTA, 30mM NaF, Na_3VO_4 0.2mM, Okadaic acid 1nM, $Na_4P_2O_7$ 5mM,1mM PMSF) supplemented with protease inhibitors (Complete; Roche 11697498001) at 4°C using a glass Dounce homogenizer. The presence of MAO-B in the homogenates was confirmed by western blot and the absence of pathological a-syn aggregates by Alphalisa.

Radiobinding assay using [^{3}H]ACI-12589 for determination of the dissociation constant (K_d) on AD brain homogenates

To determine the dissociation constant (K_d) of [^{3}H]ACI-12589 in AD brain homogenates, the AD insoluble fraction, prepared as described above, was incubated with [^{3}H]ACI-12589 or [^{3}H]PiB at different concentrations, ranging from 2nM to 400nM or 2nM to 50nM, respectively. The reaction was performed in assay buffer (50mM Tris pH 7.5 in 0.9% NaCl, 0.1% BSA) and incubated for two hours at RT. Samples in duplicate were then filtered under vacuum in GF/C filter plates (PerkinElmer) to trap the aggregates with the bound radioligand and washed five times with ice-cold 50mM Tris pH7.5. The GF/C filters were then dried and scintillation liquid (UltimateGold, PerkinElmer) was added in each well. The filters were analyzed on a Microbeta2 scintillation counter (PerkinElmer). Co-incubation with non-radiolabeled ACI-12589 or PiB at 1µM was used to determine the level of non-specific binding. Specific binding was calculated by subtracting the non-specific signal from the total signal. K_d values were calculated by nonlinear regression, using a one site specific binding model using GraphPad Prism v7.

Radiobinding assay using [^{3}H]L-deprenyl for determination of the inhibition constant (K_i) on brain homogenates containing MAO-B

Healthy control brain homogenates containing MAO-B, prepared as described above, were incubated with a tritiated MAO-B inhibitor ([^{3}H]L-deprenyl) at 10nM and increasing concentrations of selected non-radiolabelled compounds in the range of 400pM to 2µM for two hours at RT. The reaction was performed in assay buffer (50mM Tris pH 7.5 in 0.9% NaCl, 0.1% BSA) and incubated for two hours at RT. Samples in duplicate were then filtered under vacuum in GF/C filter plates (PerkinElmer) to trap the aggregates with the bound radioligand and washed five times with ice-cold 50mM Tris pH7.5. The GF/C filters were then dried and scintillation liquid (UltimateGold, PerkinElmer) was added in each well. The filters were analyzed on a Microbeta2 scintillation counter (PerkinElmer). Non-specific signal was determined with an excess of non-radiolabelled reference ligand (2µM) and specific binding was calculated by subtracting the non-specific signal from the total signal. Competition was calculated as percent, where 0% was defined as the specific binding in the presence of vehicle

and 100% as the values obtained in the presence of excess of the non-radiolabelled reference ligand. K_i values were calculated in GraphPad Prism v7 by applying a nonlinear regression curve fit using a one site, specific binding model using GraphPad Prism v7. Independent measurements were performed with two replicates.

Kinetic analyses:

Measured radioactivity concentration in whole blood and plasma were interpolated to a two-second time grid. After preliminary analysis of several models for the parent fraction, the measured parent fraction values were fitted using $\boldsymbol{f(t) = (1 + \alpha t^2)^{-\beta}}$, where $\boldsymbol{f}$ is the parent fraction and $\boldsymbol{\alpha}$ and $\boldsymbol{\beta}$ are free model parameters[3] as this model provided adequate description of the measured data. The parent fraction model fits were subsequently multiplied to the plasma curve to generate metabolite corrected arterial input functions. The input functions were used to apply several pharmacokinetic models to the time-activity curves (one- and two-tissue compartment model (1-2TCM), Logan graphical analysis and Ichise Multilinear Graphical Analysis (MA1)).

The 1TCM could not adequately describe the measured TACs. The 2TCM resulted in satisfactory model fits and systemically lower Akaike Information Criteria scores than the 1TCM, but for 26 of the total 494 regional TACs, the model parameters were not properly identified (typically k_4 approached 0, resulting in unrealistically high values of distribution volume (V_T)). Logan and MA1 could both fit all of the evaluated TACs and provided V_T estimates that were both in high agreement with each other (0.6±3.2% difference), and in good correspondence with V_T from 2TCM in all brain regions for which 2TCM provided reasonable V_T estimates. For this reason, the Logan plot was selected as the model of choice.

The simplified reference tissue model and the Logan graphical analysis with reference tissue were also evaluated. As reference tissue, the occipital and cerebellar cortices were used. Binding potential with respect to non-displaceable uptake (BP_{ND}) derived either using SRTM or Logan ref correlated with both distribution volume ratios derived using the respective V_T values, and with the actual V_T from Logan plot (Fig. 3d). Finally, to facilitate simplified acquisition protocols, SUV ratios were calculated for three different time windows (30-50, 50-

70, and 60-90-min post radioligand injection), and compared to other outcome reference-region based outcome measures. Good correspondence was observed for all time windows, and 60-90 min post injection was selected for subsequent clinical scans.

Radiosynthesis of [^{18}F]ACI-12589

[^{18}F]Fluoride was produced by a cyclotron (GE PETtrace 800) by irradiating [^{18}O]H_2O via a (p,n) reaction. The [^{18}F]fluoride (aq.) was transferred to a hot cell containing a Neptis Perform synthesis module (Optimized Radiochemical Applications) for cassette based automated radiosynthesis. The [^{18}F]fluoride was trapped on a quaternary methyl ammonium (QMA) cartridge (K-922, ABX, CO_3^{2-} preconditioned) and [^{18}O]H_2O was removed. The activity was eluted into the reaction vial using 1.0 mL of $Bu_4NH_2PO_4$ (20 mM) dissolved in 50% MeCN in H_2O. The eluted mixture was dried via azeotropic distillation by heating the reaction vial to 110 °C under N_2-flow with 3 additions of MeCN over 15 min, until the reactor was dried. The chemical precursor ACI-15051, dissolved in dry DMSO was added to the dried [^{18}F]fluoride mixture and the reaction was heated for 10 min. The reaction was cooled and then quenched by addition of 6 mL of H_2O. The diluted reaction crude was injected on a semi-preparative HPLC column (Agilent Zorbax eclipse C18, 250 x 9,4 mm, 5 µm, equipped with a 4,6 x 12,5 mm guard column) with an eluent consisting of 25% MeCN in 20 mM citrate buffer (pH 4.4) at a flow rate of 6 ml/min. The fraction corresponding to the desired product was collected for 90 s and diluted by ca 65 mL H_2O. The diluted fraction was extracted using a C18-cartridge (Sep Pak C18 light, Waters) previously preconditioned with 5 mL EtOH and washed with 5 mL H_2O and flushed with air. The C18-cartridge was washed with 10 mL of H_2O and then eluted using 2.0 mL of 50% EtOH in H_2O into a transfer vial containing 10 mL NaCl solution (9 mg/mL). The C18-cartridge was then rinsed with 6 mL of NaCl solution (9 mg/mL) into the transfer vial and the complete content was subsequently transferred to the final product vial via a sterile filter (Cathivex-GV 0.22 µm, Merck Millipore). The automated radiosynthesis manufacturing process was fully validated and achieved an average of 9,3 ± 4,0 GBq of radioactive product per batch with a radiochemical yield of 25.3 ± 4.5% (n = 25).

Quality control – HPLC and TLC

The product was analysed by HPLC (pump, autosampler, column compartment, UV-detector: Dionex UltiMate 3000, ThermoFischer Scientific, radiodetector: NaI-detector (B-FC-3200) and flowcount (Bioscan B-FC-1000) Eckert & Ziegler Radiopharma, Inc. connected to Dionex Universal Chromatography Interface (UCI-50/100).

Chromeleon version 7 (ThermoScientific) was used as chromatography system. The analytical HPLC was used for the determination of identity of product, radiochemical purity (RCP), specific radioactivity (A_S), amount of ACI-12589 and unrelated UV-active impurities. New batches of precursor also required a test synthesis with full quality control plus determination of enantiomeric purity by a chiral HPLC method. Analytical parameters used are listed in Supplementary Table 3.

TLC was used for analysis of free [^{18}F]fluoride in the product by spotting 2 µL of product onto a silica TLC-plate (TLC Silica gel 60 F_{254} Aluminium TLC plate, Merck) which was then eluted using 80% MeCN in H_2O. The plates were analysed using a radio-TLC scanner (detector FC-3600, Mini-Scan B-MS-1000, flowcount BioScan FC-1000, Eckert & Ziegler) connected to Dionex Universal Chromatography Interface (UCI-50/100).

The product was analysed with respect to pH, Bu_4^+-content, residual solvents, endotoxin, sterility, radionuclide identity and radionuclidic purity according to European Pharmacopoeia (Ph. Eur.).

Blood sampling and metabolite analysis

Arterial blood samples were obtained manually at baseline and 45 seconds (s), 90 s, 135 s, 180 s, 225 s, 270 s*, 315 s, 6 min*, 8 min, 10 min*, 15 min, 20 min*, 25 min, 30 min*, 45 min, 60 min*, 75 min, 90 min* post injection (2 or 5 mL* per sample). Plasma was separated by centrifugation at 3000 x g (4400 rpm; Centrifuge 5702, Eppendorf) for 10 min, and radioactivity was measured in a γ-counter (Hidex AMG, energy window 370-1100 keV, calibration constant 0.52 Bq^{-1} and counting time 30 sec) for both whole-blood and plasma samples (200 µL each).

Determination of fraction of intact [^{18}F]ACI-12589 in plasma was carried out for samples at baseline, 4.5, 6, 10, 20, 30, 60 and 90 min as follows. Plasma samples were processed by MeCN precipitation, treating 1 mL of plasma with 1 mL of MeCN. After vortex mixing and centrifugation at 3000 x g (4400 rpm) for 10 min, 1 mL of the supernatant was diluted with 1 mL Milli-Q water and analyzed by HPLC (pump, injector, UV-detector, and fraction collector: Ultimate 3000, Thermo Scientific; BGO radioactivity detector: Bioscan). The sample was injected on a Phenomenex Luna 10 µm C18(2) 100Å (10 x 250 mm) and eluted with a mobile phase of 60% MeOH in H_2O containing 0.2% of triethylamine at a flow rate of 4 mL/min. The eluent from the column was collected in 15 different fractions (4 mL/fraction), each fraction was measured in a γ-counter to determine the percentage of intact parent compound. The percentage of parent compound was calculated by dividing the radioactivity from the fractions representing parent compound by the total sum of all fractions (Supplementary Figure 3).

For the determination of plasma free fraction 200 µL of plasma spiked with formulated [^{18}F]ACI-12589 (300 -500 kBq) were pipetted in duplicates into ultrafiltration units (Amicon Centrifree 30, Millipore) and centrifuged for 20 min at 3000 x g (4400 rpm). The radioactivity of the ultrafiltrate and the filtration unit were measured (dose calibrator, Capintec 120-CRC), and the plasma free fraction could be calculated.

Suppl. Figure 1.
Structure and synthesis of [18F] ACI-12589

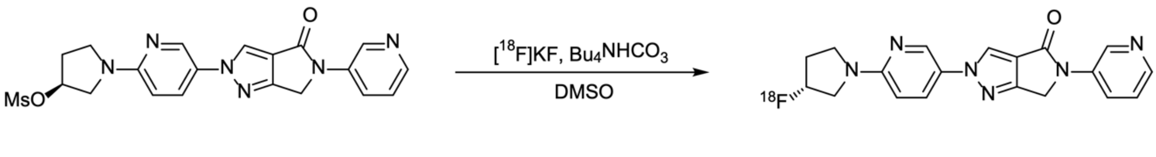

ACI-15051

[^{18}F]-ACI-12589

Suppl. Figure 2.

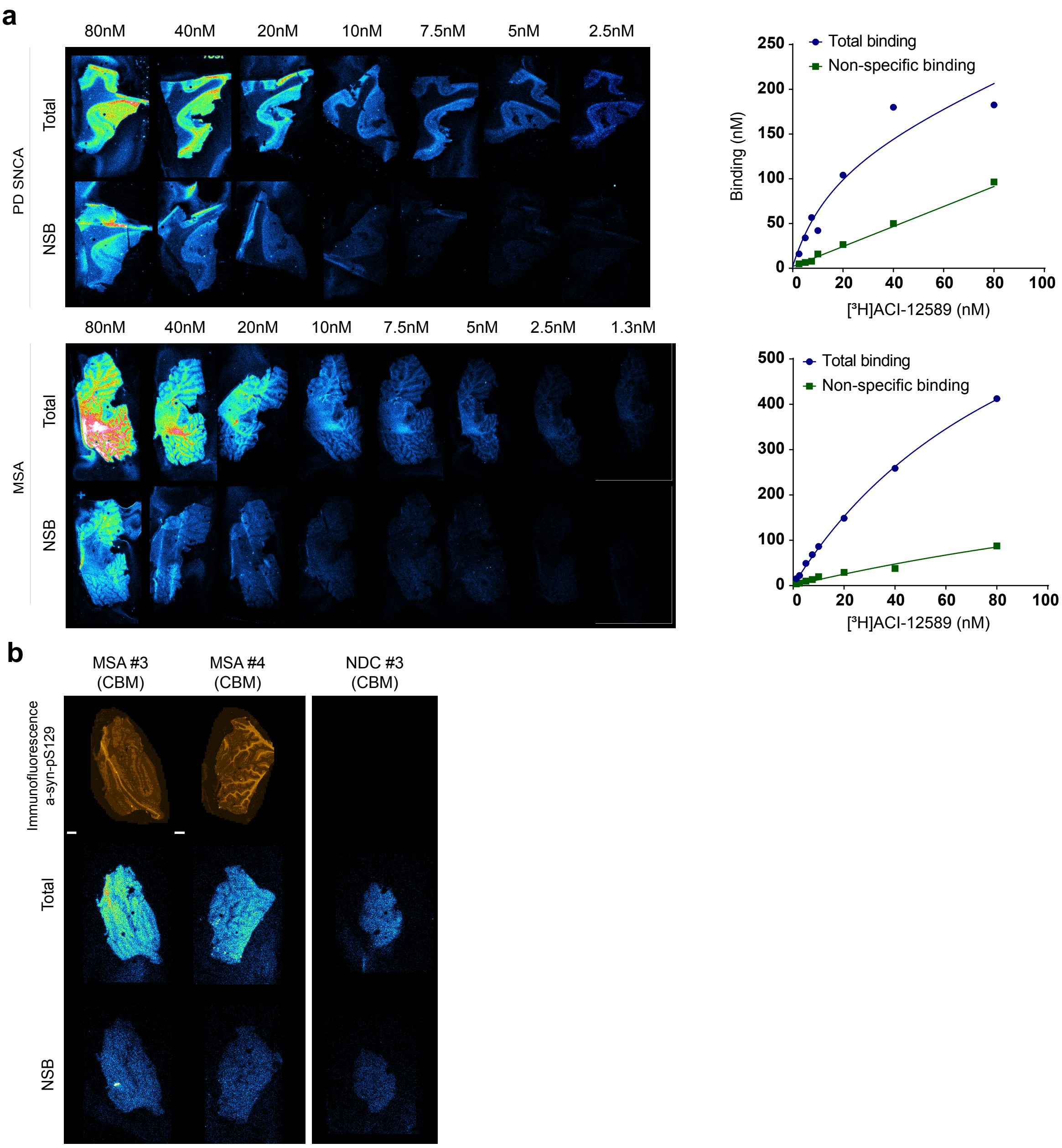

Autoradiography and saturation binding experiments.

[^{3}H]ACI-12589 and [^{18}F]ACI-12589 display specific binding in human brain tissues containing α-synuclein *ex vivo*. a) Saturation binding studies with [^{3}H]ACI-12589 on human brain tissue sections from from a familial PD (PD SNCA, top) and a MSA case (bottom). Left, autoradiographic detection of [^{3}H]ACI-12589 binding in brain tissue sections. Total: total binding (2.5 nM - 80 nM); NSB: Non-specific binding, as defined by residual binding in the presence of 2 or 5 µM unlabelled ACI-12589. Right, total and non-specific binding of [^{3}H]ACI-12589 with increasing concentration of ligand on the x-axis. Fitting the data was performed with non-linear regression analysis, using a one-site specific binding model in GraphPad Prism. Staining of adjacent sections with α-syn-pS129. PD SNCA: PD with SNCA G51D mutation; MSA: multiple system atrophy; Scale bar, 2 mm. (b) Autoradiography with [^{3}H]ACI-12589 in MSA tissue from the cerebellum brain region and brain region-matched control tissue. Total: total binding (10 nM); NSB: Non-specific binding (5 µM), as defined by residual

binding in the presence of 5 µM unlabelled ACI-12589. Immunofluorescence with α-syn-pS129 antibody (top panels) on the same sections. CBM: Cerebellum; Scale bar, 2 mm.

Suppl. Figure 3.

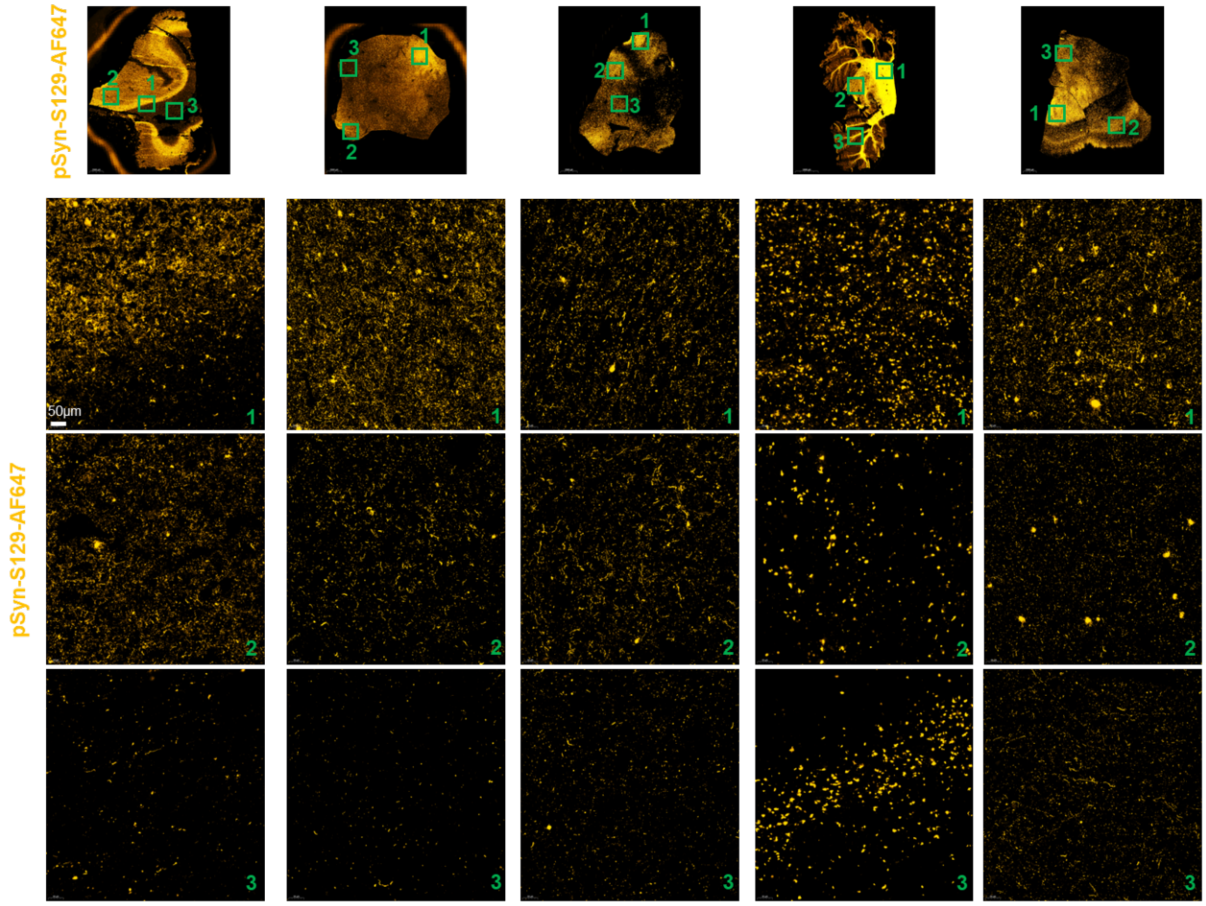

High resolution images from Figure 1c.
Immunofluorescent staining with a-syn-pS129 for cases shown in Fig. 1c. Zoomed-in images for different regions of each case are shown in the bottom panels, as indicated by green rectangles (1-3). Scale bar, 50 µm.

Suppl. Figure 4.

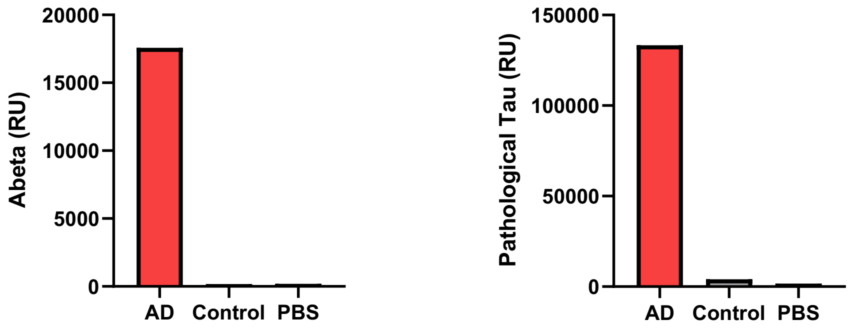

Confirmation of the presence of pathological β-Amyloid and Tau in tissue donors in Fig. 2a, c. a) Biochemical characterization of AD brain homogenates used to measure specific binding in Fig. 2a, head-to-head with healthy control homogenates and PBS. β-Amyloid and pathological tau levels were measured by AlphaLisa. RU: relative units.

Suppl. Figure 5.

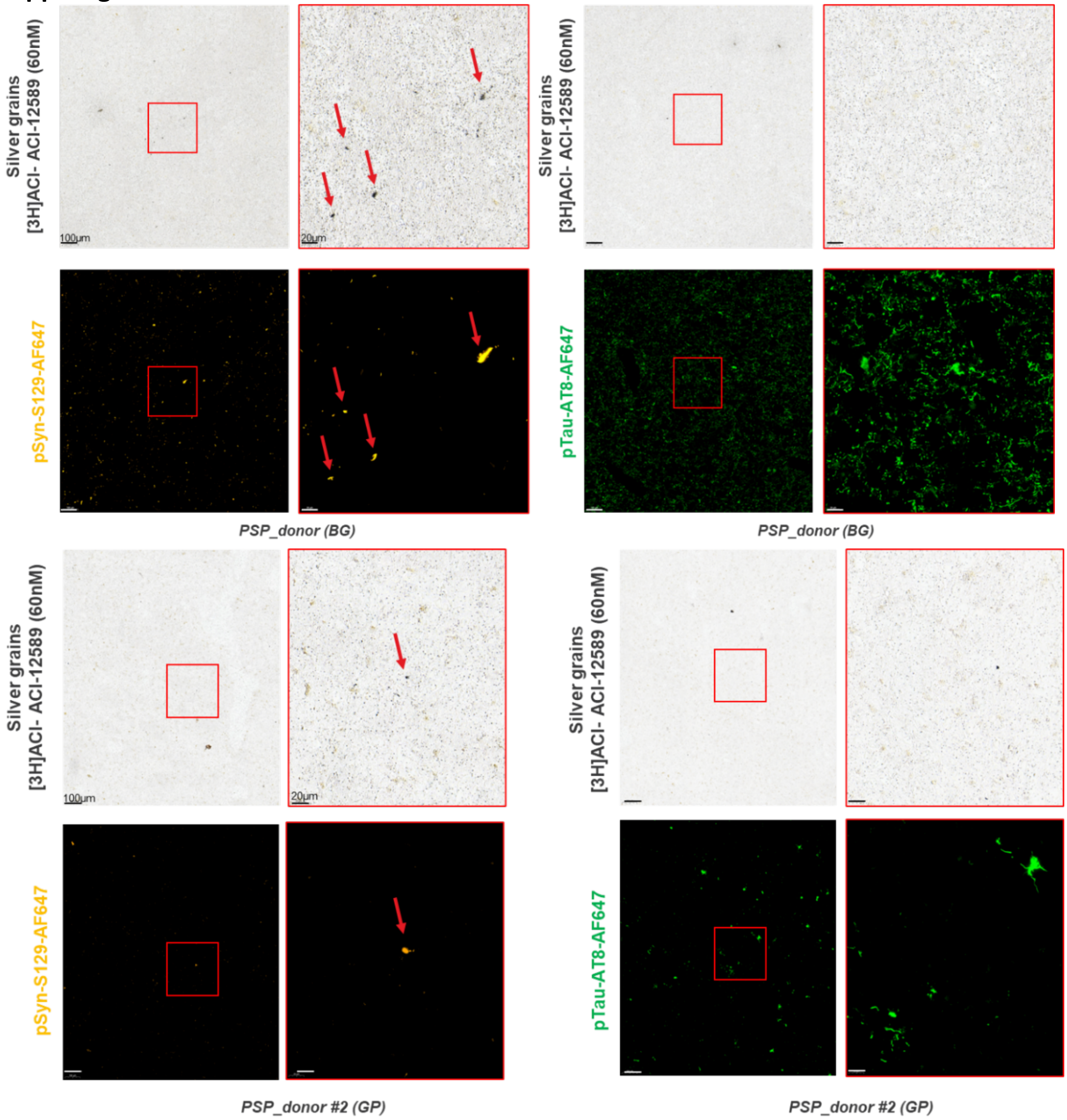

Immunofluorescence with α-syn-pS129 antibody or pTau-AT8 antibody in brain tissue sections from two PSP cases showing α-syn pathology (bottom left panels) or tau pathology (bottom right panels). High-resolution ARG with [^{3}H]ACI-12589 (60nM) in the same sections, showing co-labeling of α-syn aggregates (top left panels, red arrows) and absence of binding to tau aggregates (top right panels). Red rectangle in the zoomed-out image (scale bar, 100 µm) indicates the region of magnification for the zoomed-in image (scale bar, 20µm).

Suppl. Figure 6.

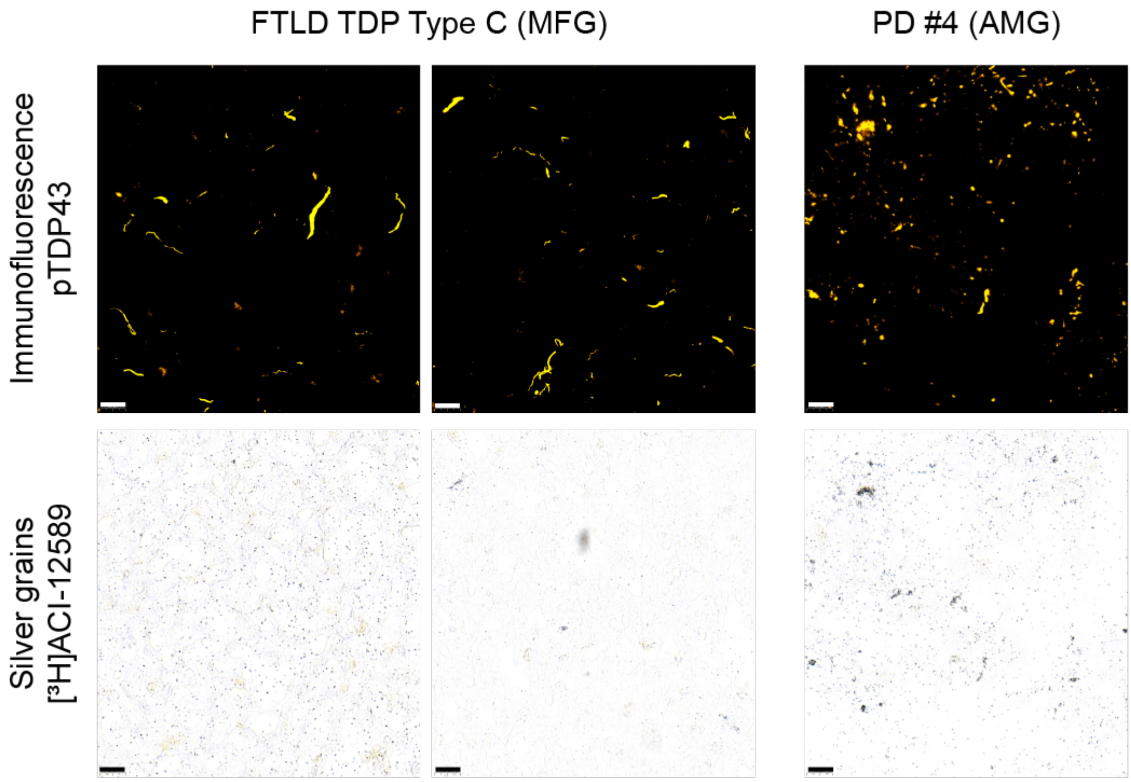

TDP-43 autoradiography.

Assessment of target engagement of [^{3}H]ACI-12589 on FTLD TDP-43 type C tissue containing pathological TDP-43 aggregates. High-resolution ARG with [^{3}H]ACI-12589 (60nM) on FTLD TDP-43 type C tissue and PD tissue. Immunofluorescence staining with phospho-TDP-43 (pTDP43) antibody on the same FTLD TDP-43 type C tissue labelling TDP-43 aggregates or with α-syn-pS129 antibody on the PD section, labeling α-syn aggregates. No accumulation of silver grains on TDP-43 aggregates with [^{3}H]ACI-12589. MFG: Middle Frontal Gyrus, AMG: Amygdala; Scale bar, 20 µm.

Suppl. Figure 7.

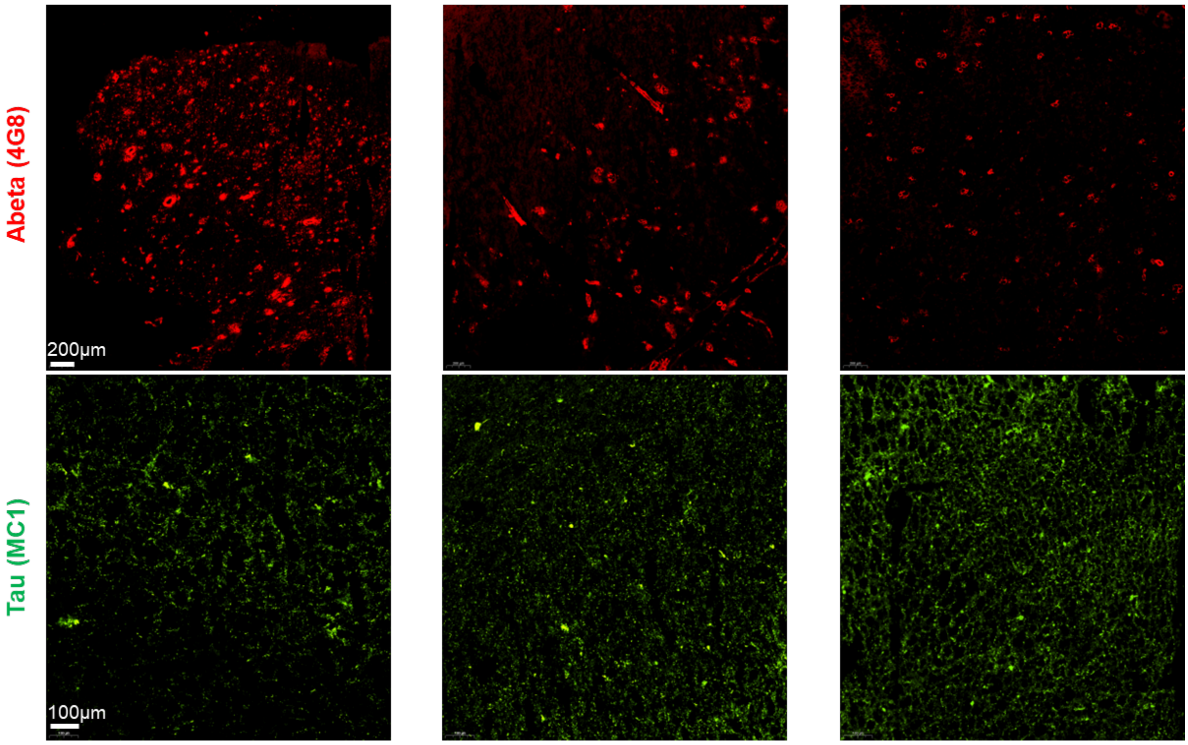

β-Amyloid and Tau staining of the AD donor with mixed a-syn pathology (Fig. 2c)
Immunofluorescence for Aβ (using mAb, 4G8) and Tau (using mAb, MC1) pathology in AD tissue donor sections also used in the data presented in Fig. 2c. Scale bar in top panels, 200 µm. Scale bar in bottom panels, 100 µm.

Supplementary Table 1

Evaluation of Kd and Bmax of [3H]ACI-12589 across different donors by autoradiography

Donor	MSA	Familial PD	Idiopathic PDD	Idiopathic PDD
**Bmax[1] (nM)**	~350	133	80	14
**Kd[1] (nM)**	28	17	65	38
**R^2**	0.92	0.76	0.97	0.87
**95% Confidence Interval (nM)**	19 – 42	3 - 102	36 - 152	11 - 357

Supplementary Table 2

Evaluation of Kd and Bmax of [3H]ACI-12589 across different donors by micro-radiobinding or autoradiography

Idiopathic PD	Technique	Kd (nM)	R^2	95% CI	# of repetitions
**Donor #1 (temporal cortex)**	Radiobinding	21	0.94	15 to 31	2
**Donor #2 (amygdala)**	Radiobinding	5.0	0.93	3.5 to 6.8	1
**Donor #3 (amygdala)**	Radiobinding	26	0.94	16 to 45	1
**Donor #4 (amygdala)**	Radiobinding	24	0.81	15 to 39	3
**Donor #1 (amygdala)**	Radiobinding	32	0.73	20 to 53	5
**Donor #5 (temporal cortex)**	Radiobinding	56	0.85	40 to 81	8
**Donor #6 (amygdala)**	Radiobinding	27	0.92	15 to 48	1
**Donor #7 (amygdala)**	Radiobinding	41	0.90	23 to 88	1
**Donor #8 (amygdala)**	Autoradiography	65	0.97	36 to 152	1
**Donor #9 (amygdala)**	Autoradiography	38	0.87	11 to 357	1

Supplementary Figure 8

***In vitro* off-target binding assay.** In each assay, only results showing effects larger than 50% are considered relevant. Effects in the 25%-30% are not considered significant and mostly attributable to variability of the signal around the control level.

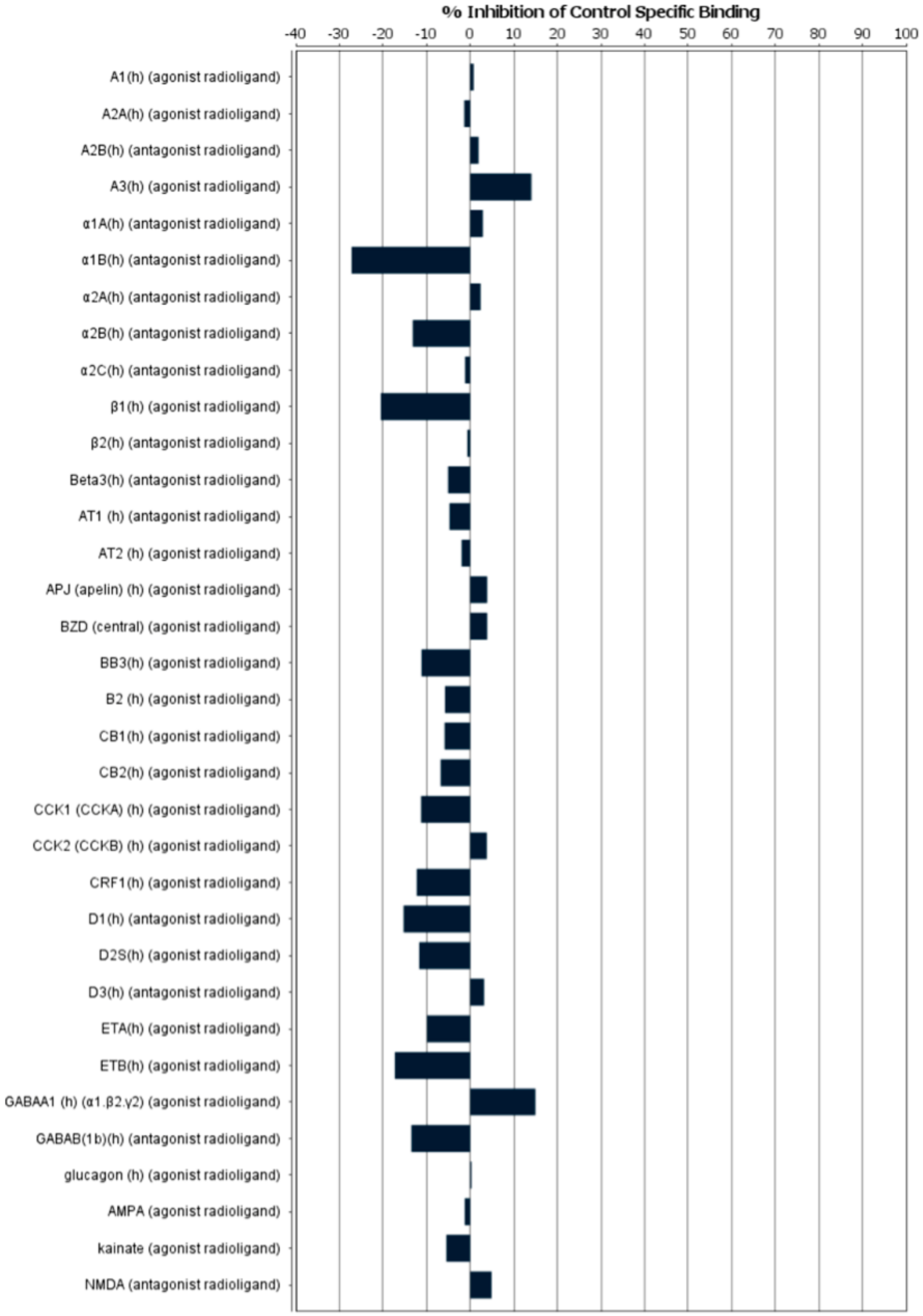

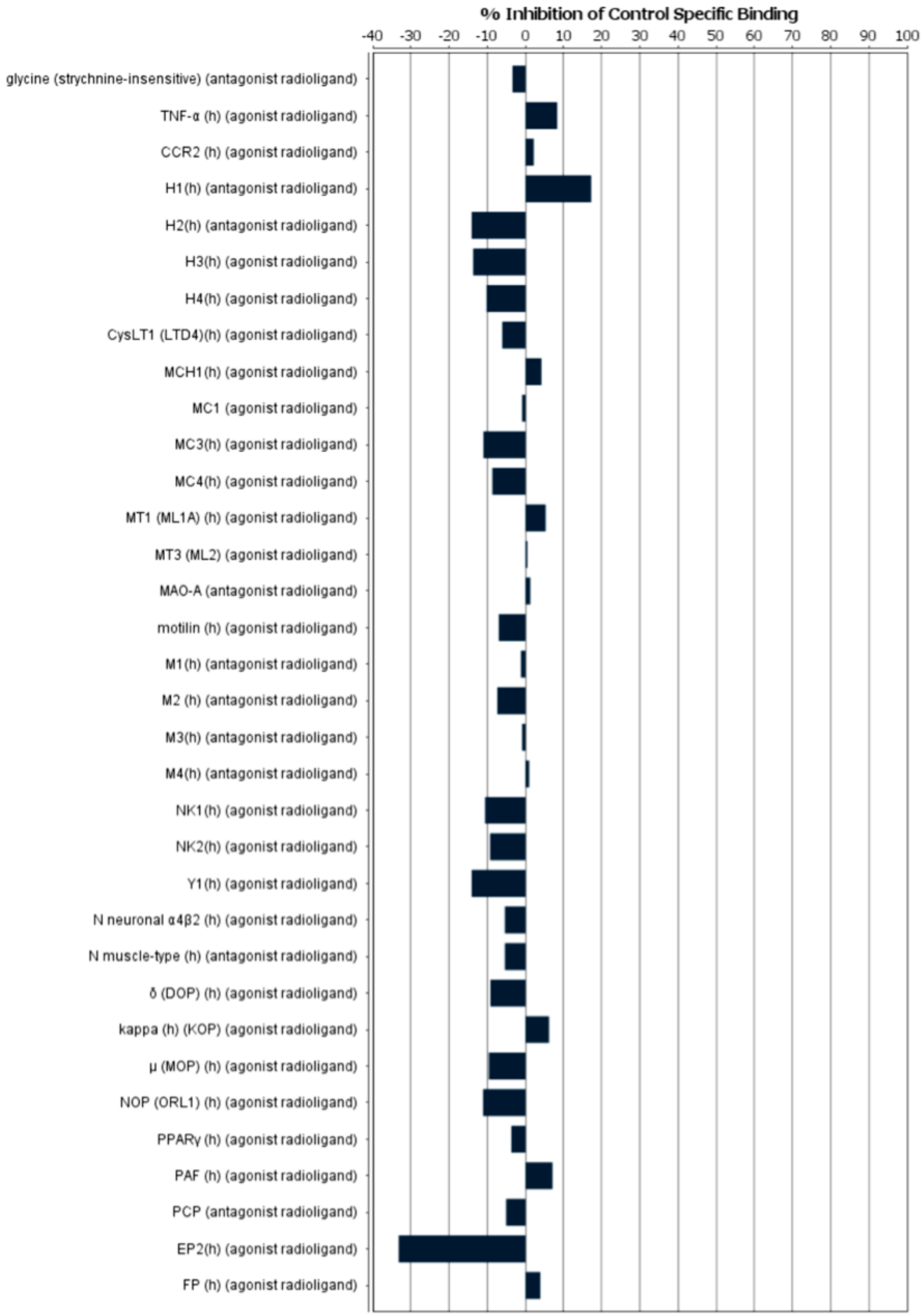

% Inhibition of Control Specific Binding
-40 -30 -20 -10 0 10 20 30 40 50 60 70 80 90 100
glycine (strychnine-insensitive) (antagonist radioligand)
TNF-α (h) (agonist radioligand)
CCR2 (h) (agonist radioligand)
H1(h) (antagonist radioligand)
H2(h) (antagonist radioligand)
H3(h) (agonist radioligand)
H4(h) (agonist radioligand)
CysLT1 (LTD4)(h) (agonist radioligand)
MCH1(h) (agonist radioligand)
MC1 (agonist radioligand)
MC3(h) (agonist radioligand)
MC4(h) (agonist radioligand)
MT1 (ML1A) (h) (agonist radioligand)
MT3 (ML2) (agonist radioligand)
MAO-A (antagonist radioligand)
motilin (h) (agonist radioligand)
M1(h) (antagonist radioligand)
M2 (h) (antagonist radioligand)
M3(h) (antagonist radioligand)
M4(h) (antagonist radioligand)
NK1(h) (agonist radioligand)
NK2(h) (agonist radioligand)
Y1(h) (agonist radioligand)
N neuronal α4β2 (h) (agonist radioligand)
N muscle-type (h) (antagonist radioligand)
δ (DOP) (h) (agonist radioligand)
kappa (h) (KOP) (agonist radioligand)
μ (MOP) (h) (agonist radioligand)
NOP (ORL1) (h) (agonist radioligand)
PPARγ (h) (agonist radioligand)
PAF (h) (agonist radioligand)
PCP (antagonist radioligand)
EP2(h) (agonist radioligand)
FP (h) (agonist radioligand)

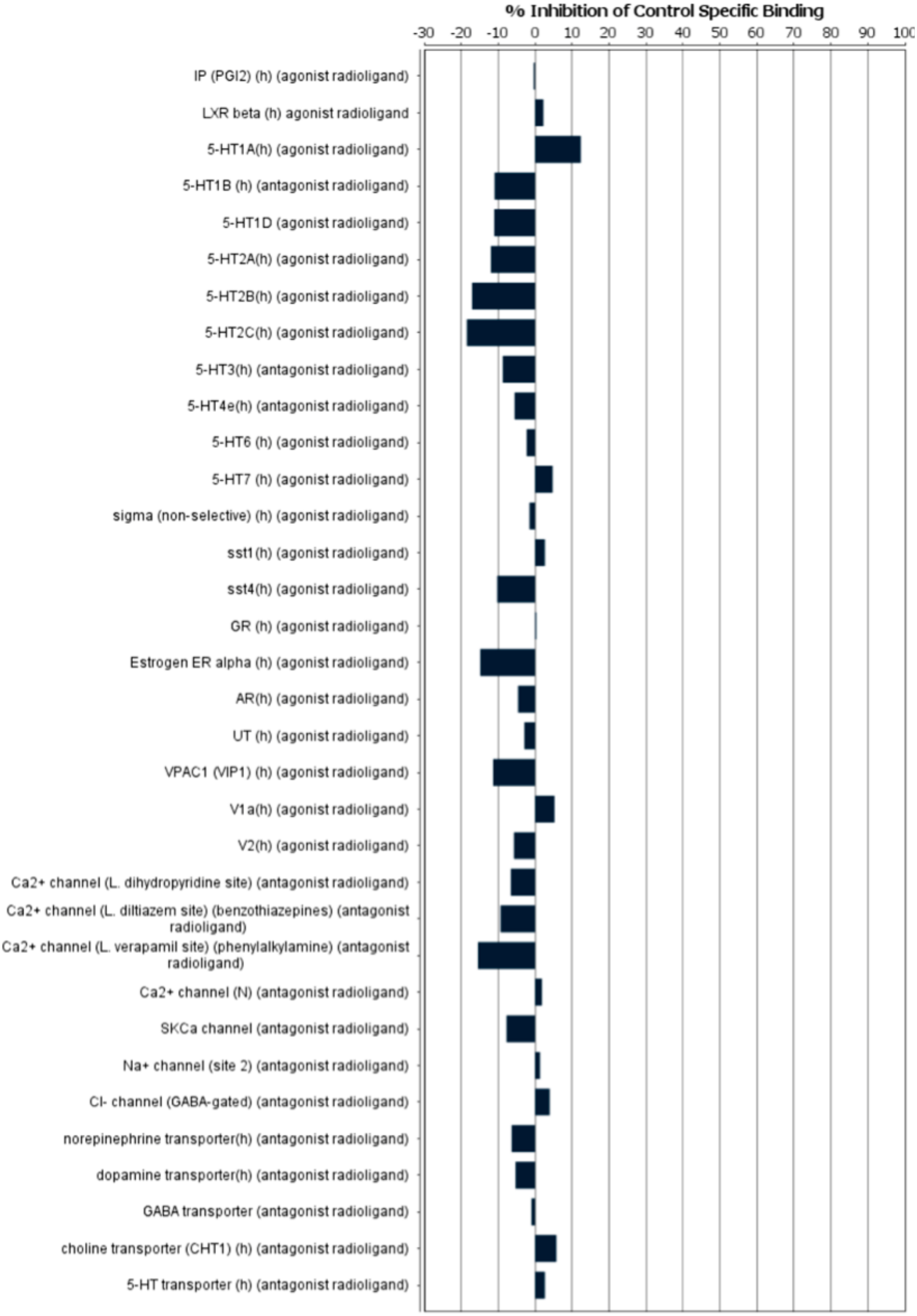
% Inhibition of Control Specific Binding
-30 -20 -10 0 10 20 30 40 50 60 70 80 90 100
IP (PGI2) (h) (agonist radioligand)
LXR beta (h) agonist radioligand
5-HT1A(h) (agonist radioligand)
5-HT1B (h) (antagonist radioligand)
5-HT1D (agonist radioligand)
5-HT2A(h) (agonist radioligand)
5-HT2B(h) (agonist radioligand)
5-HT2C(h) (agonist radioligand)
5-HT3(h) (antagonist radioligand)
5-HT4e(h) (antagonist radioligand)
5-HT6 (h) (agonist radioligand)
5-HT7 (h) (agonist radioligand)
sigma (non-selective) (h) (agonist radioligand)
sst1(h) (agonist radioligand)
sst4(h) (agonist radioligand)
GR (h) (agonist radioligand)
Estrogen ER alpha (h) (agonist radioligand)
AR(h) (agonist radioligand)
UT (h) (agonist radioligand)
VPAC1 (VIP1) (h) (agonist radioligand)
V1a(h) (agonist radioligand)
V2(h) (agonist radioligand)
Ca2+ channel (L. dihydropyridine site) (antagonist radioligand)
Ca2+ channel (L. diltiazem site) (benzothiazepines) (antagonist radioligand)
Ca2+ channel (L. verapamil site) (phenylalkylamine) (antagonist radioligand)
Ca2+ channel (N) (antagonist radioligand)
SKCa channel (antagonist radioligand)
Na+ channel (site 2) (antagonist radioligand)
Cl- channel (GABA-gated) (antagonist radioligand)
norepinephrine transporter(h) (antagonist radioligand)
dopamine transporter(h) (antagonist radioligand)
GABA transporter (antagonist radioligand)
choline transporter (CHT1) (h) (antagonist radioligand)
5-HT transporter (h) (antagonist radioligand)

Suppl. Figure 9.

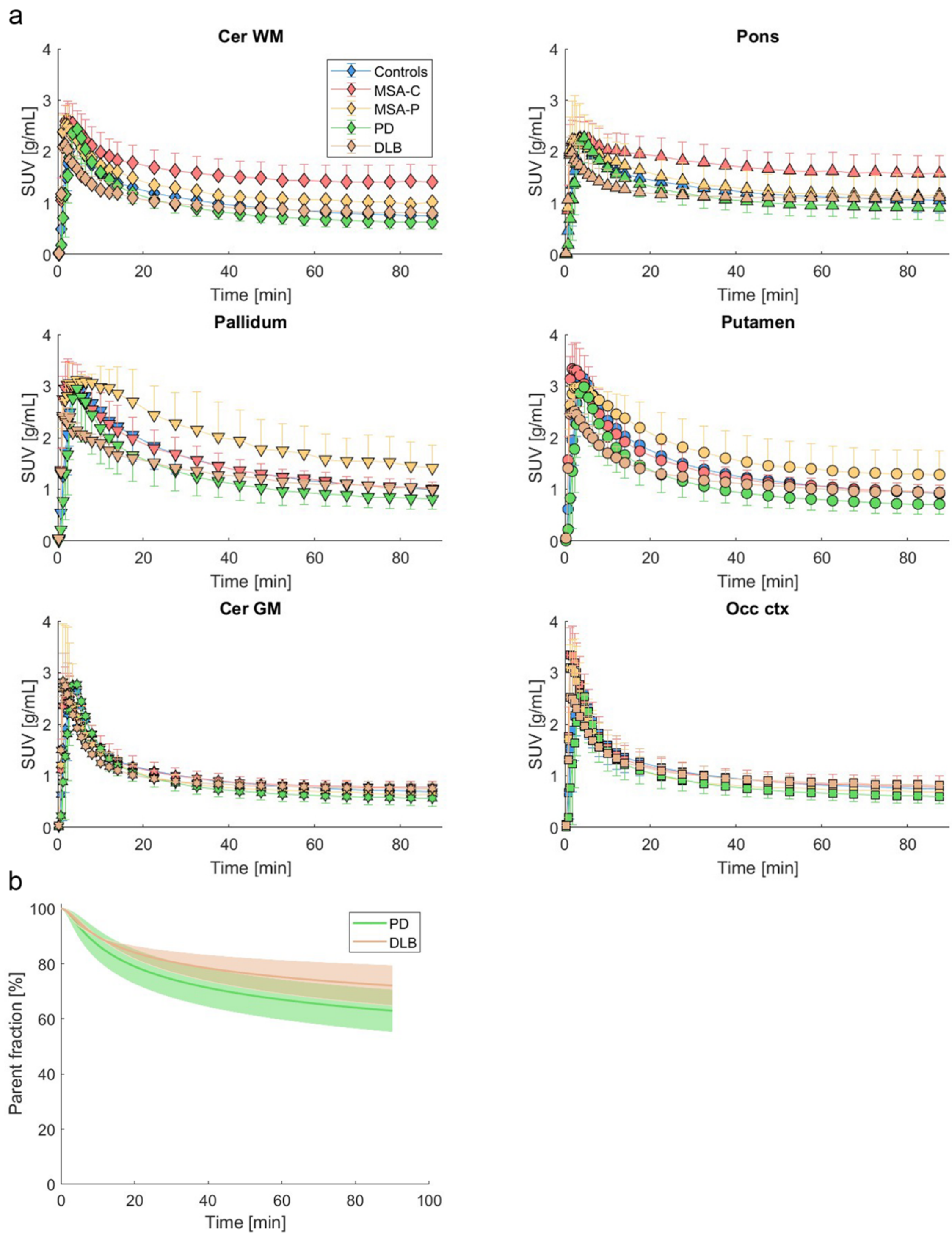

Time activity curves and parent fractions.

a) Time activity curves for all participating groups in cerebellar white matter (Cer WM), pons, pallidum, putamen, cerebellar grey matter (Cer GM) and occipital cortex (Occ Ctx). b) parent fractions in PD and DLB participants. Ctrl n = 7, DLB n = 2, MSA-C n = 6, MSA-P n = 2, PD n = 5.

Suppl. Figure 10.

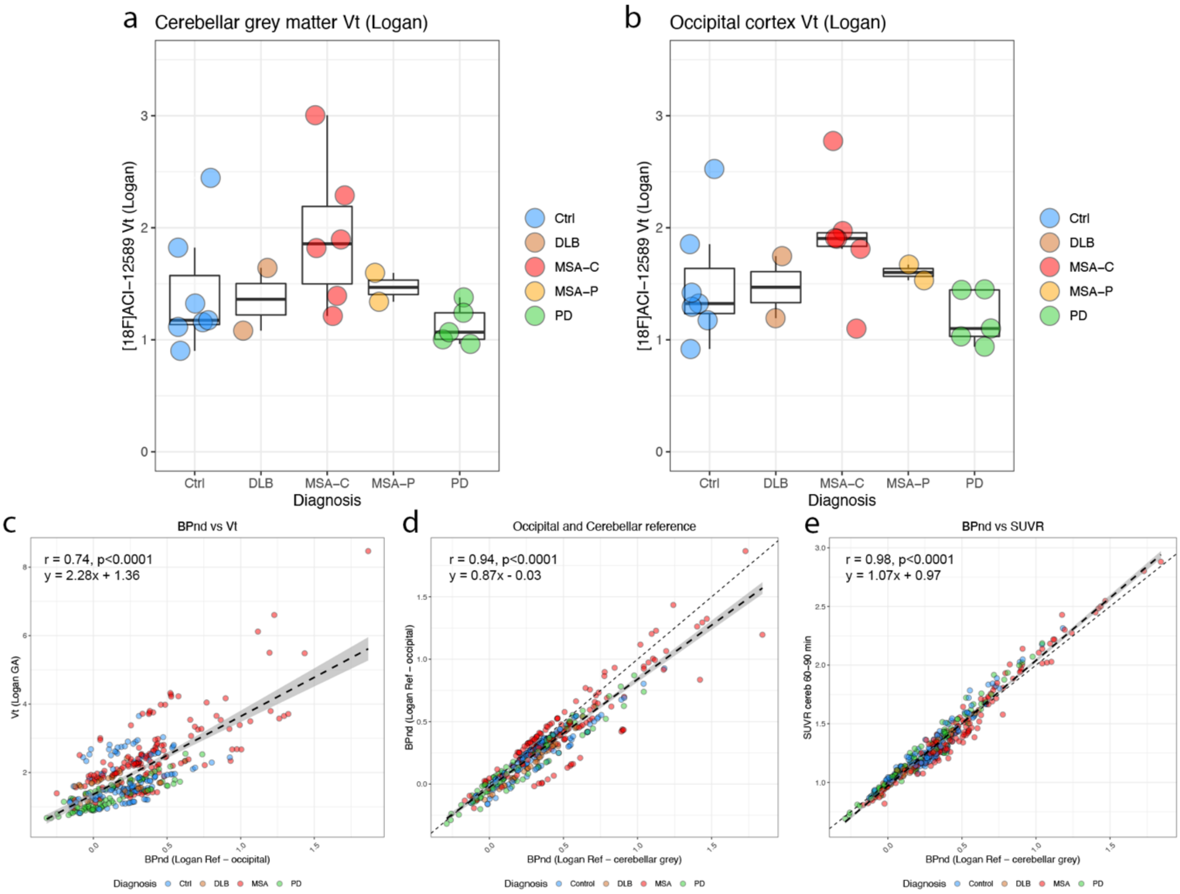

Reference region data and kinetic modelling

Regional ACI-12589 V_T values using Logan graphical analysis with blood input in potential cerebral reference regions and correlations of kinetic models. a) V_Ts in the cerebellar grey matter. Kruskal-Wallis rank sum test p=0.14. b) V_Ts in the occipital cortex. Kruskal-Wallis rank sum test p=0.11. c) V_T values derived using logan graphical analysis with blood input function (y-axis) plotted against BP_{ND} values derived using Logan reference (occipital cortex; x-axis). R-value 0.74 (95% C.I. [0.69 – 0.78]) derived using Pearson's correlation (t = 21.557, df = 394, p-value < 2.2e-16). d) BP_{ND} values from Logan reference models comparing an occipital reference region (y-axis) to a cerebellar grey matter reference region (x-axis). R-value 0.94 (95% C.I. [0.92 – 0.95]) derived using Pearson's correlation (t = 55.255, df = 423, p-value < 2.2e-16). e) Comparison of SUVR values with a cerebellar grey matter reference region in the 60-90 minute time interval with BP_{ND} values derived using a Logan reference model with a cerebellar grey matter reference region. R-value 0.98 (95% C.I. [0.976 – 0.983]) derived using Pearson's correlation (t = 103.82, df = 448, p-value < 2.2e-16). No correction for multiple comparisons was performed. Ctrl n = 7, DLB n = 2, MSA-C n = 6, MSA-P n = 2, PD n = 5. Boxplots show median, IQR (box) and whiskers (Q1 - 1.5*IQR / Q3 + 1.5*IQR or minimum/maximum value, outliers not included). BP_{ND}: non-displaceable binding potential; Ctrl: Control; DLB: Dementia with lewy bodies; MSA-C: Multiple system atrophy with a cerebellar phenotype; MSA-P: Multiple system atrophy with a parkinsonian phenotype; PD: Parkison's Disease; SUVR: standardized uptake value ratio; V_T: volume of distribution.

Suppl. Figure 11.

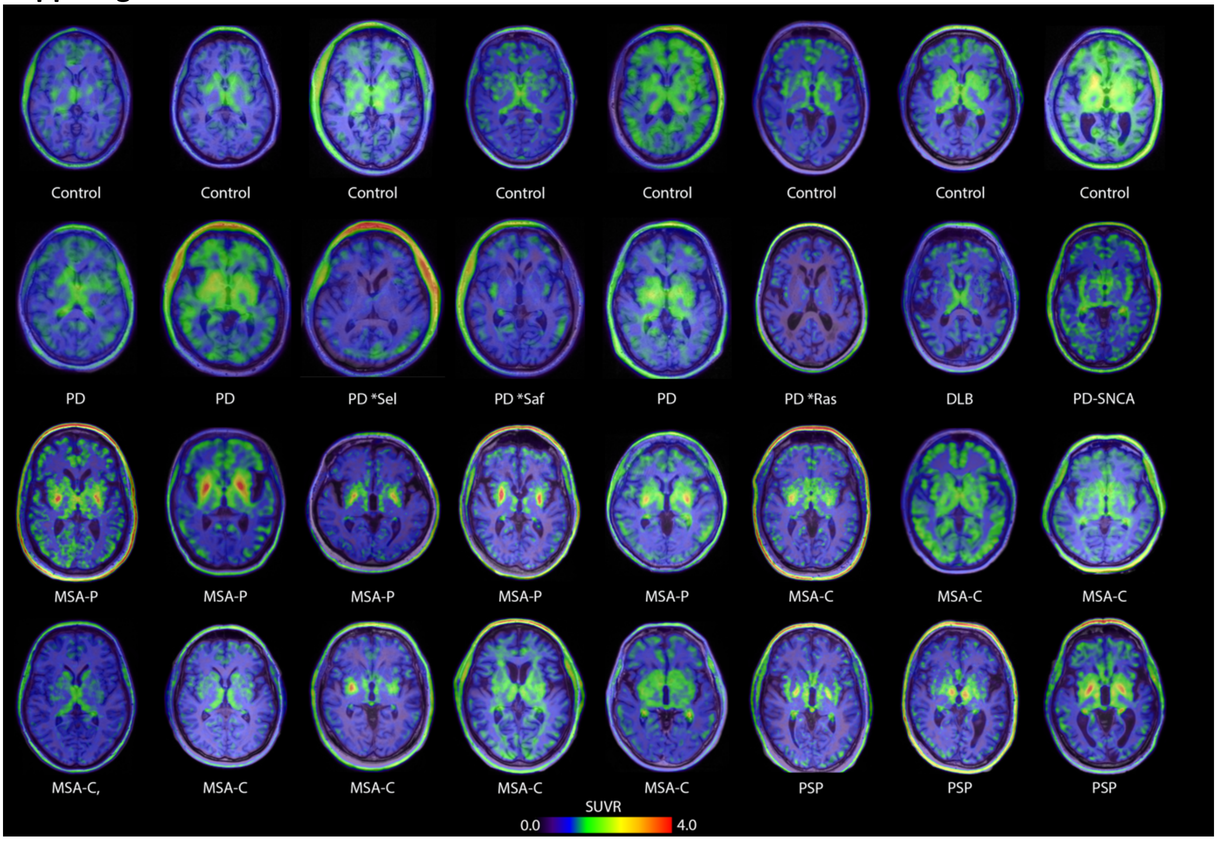

Transversal [^{18}F]ACI-12589 images at the level of the basal ganglia.

Shown are 8 controls, 7 PD patients, one DLB patient, the 13 MSA patients and 3 participants with PSP. * indicates that the participant has been taking MAO-B inhibitors (Sel – Selegiline; Saf – Safinamide; Ras – Rasagiline). DLB: Dementia with lewy bodies; MSA-C: Multiple system atrophy with a cerebellar phenotype; MSA-P: Multiple system atrophy with a parkinsonian phenotype; PD: Parkinson's Disease; PSP: Progressive supranuclear palsy; SUVR: Standardized uptake value ratio.

Suppl. Figure 12.

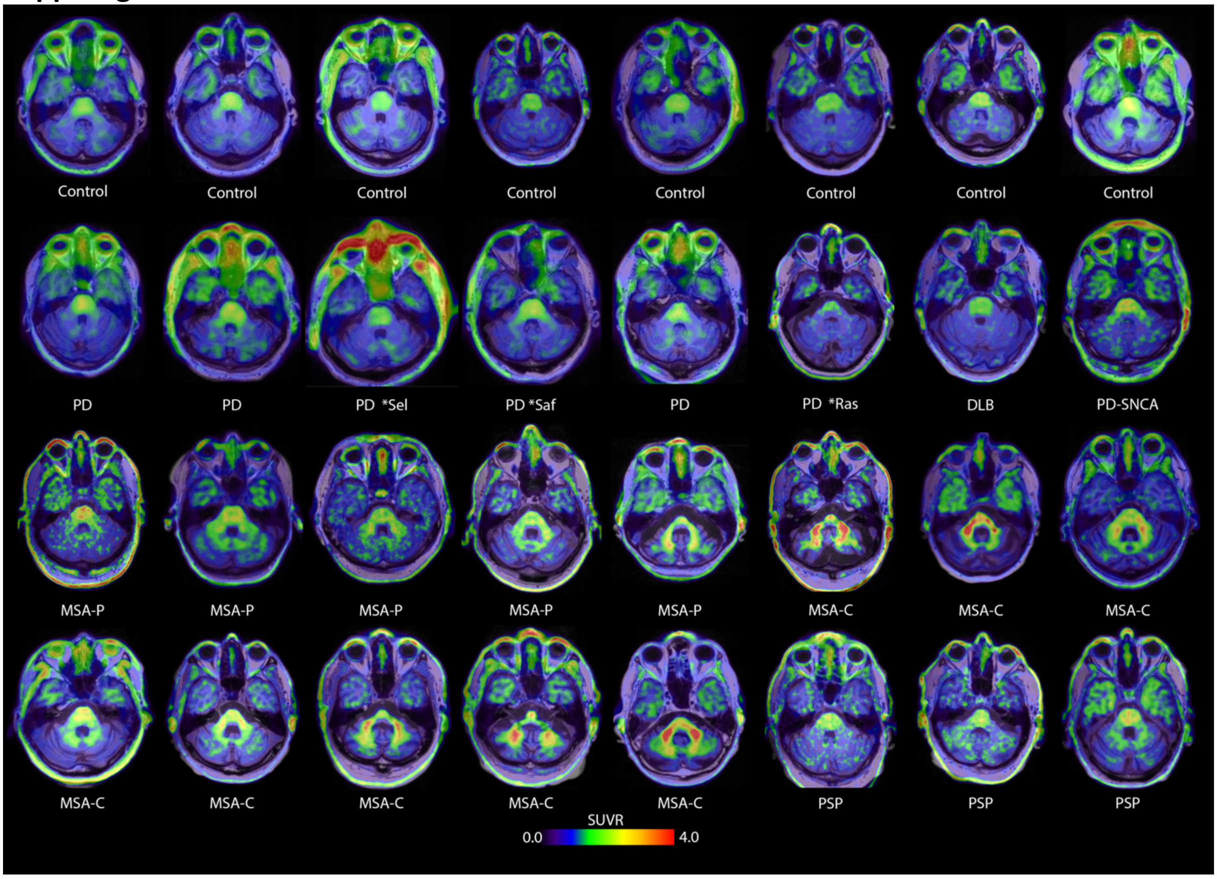

Transversal [^{18}F]ACI-12589 images at the level of the middle cerebellar peduncles. Shown are 8 controls, 7 PD patients, one DLB patient, the 13 MSA patients and 3 participants with PSP. * indicates that the participant has been taking MAO-B inhibitors (Sel – Selegiline; Saf – Safinamide; Ras – Rasagiline). DLB: Dementia with lewy bodies; MSA-C: Multiple system atrophy with a cerebellar phenotype; MSA-P: Multiple system atrophy with a parkinsonian phenotype; PD: Parkinson's Disease; PSP: Progressive supranuclear palsy; SUVR: Standardized uptake value ratio.

Suppl. Figure 13.

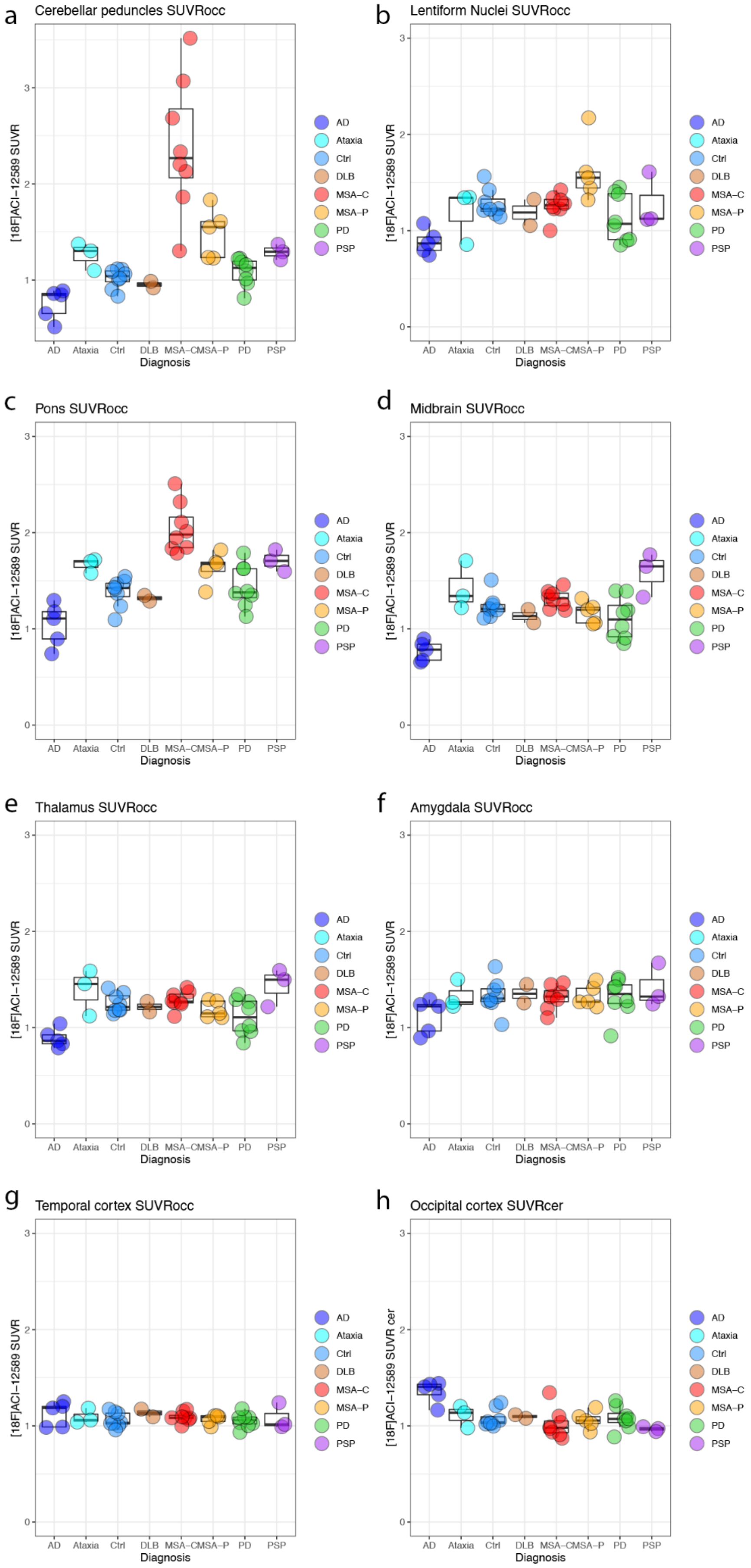

Regional retention of [^{18}F]ACI-12589 in the different diagnostic groups. Graphs in panel a) – g) shows data with an occipital cortex reference, panel h) shows the occipital cortex using a cerebellar grey matter reference region. a) middle cerebellar peduncle retention. b) retention in the lentiform nuclei (bilateral putamen and globus pallidus). Retention in the c) pons, d) midbrain, e) thalamus, f) amygdala and g) temporal lobe cortex. h) shows SUVRs in the occipital cortex using a cerebellar grey matter reference region. Boxplots show median, IQR (box) and whiskers (Q1 - 1.5*IQR / Q3 + 1.5*IQR or minimum/maximum value, outliers not included). Ctrl n = 8, DLB n = 2, MSA-C n = 8, MSA-P n = 5, PD n = 8, Ataxia n = 3, PSP n = 3, AD n = 5.
AD: Alzheimer's Disease; Ataxia: cerebellar ataxias (Friedreich Ataxia and cerebellar ataxia due to a *SAMD9L* mutation); Ctrl: control subjects; MSA-C: Multiple system atrophy with a cerebellar phenotype; MSA-P: Multiple system atrophy with a parkinsonian phenotype; PSP: Progressive supranuclear palsy; $SUVR_{cer}$: SUVR with a cerebellar grey matter reference region; $SUVR_{occ}$: SUVR with an occipital cortex reference region; SUVR: Standardized uptake value ratio.

Suppl. Figure 14.

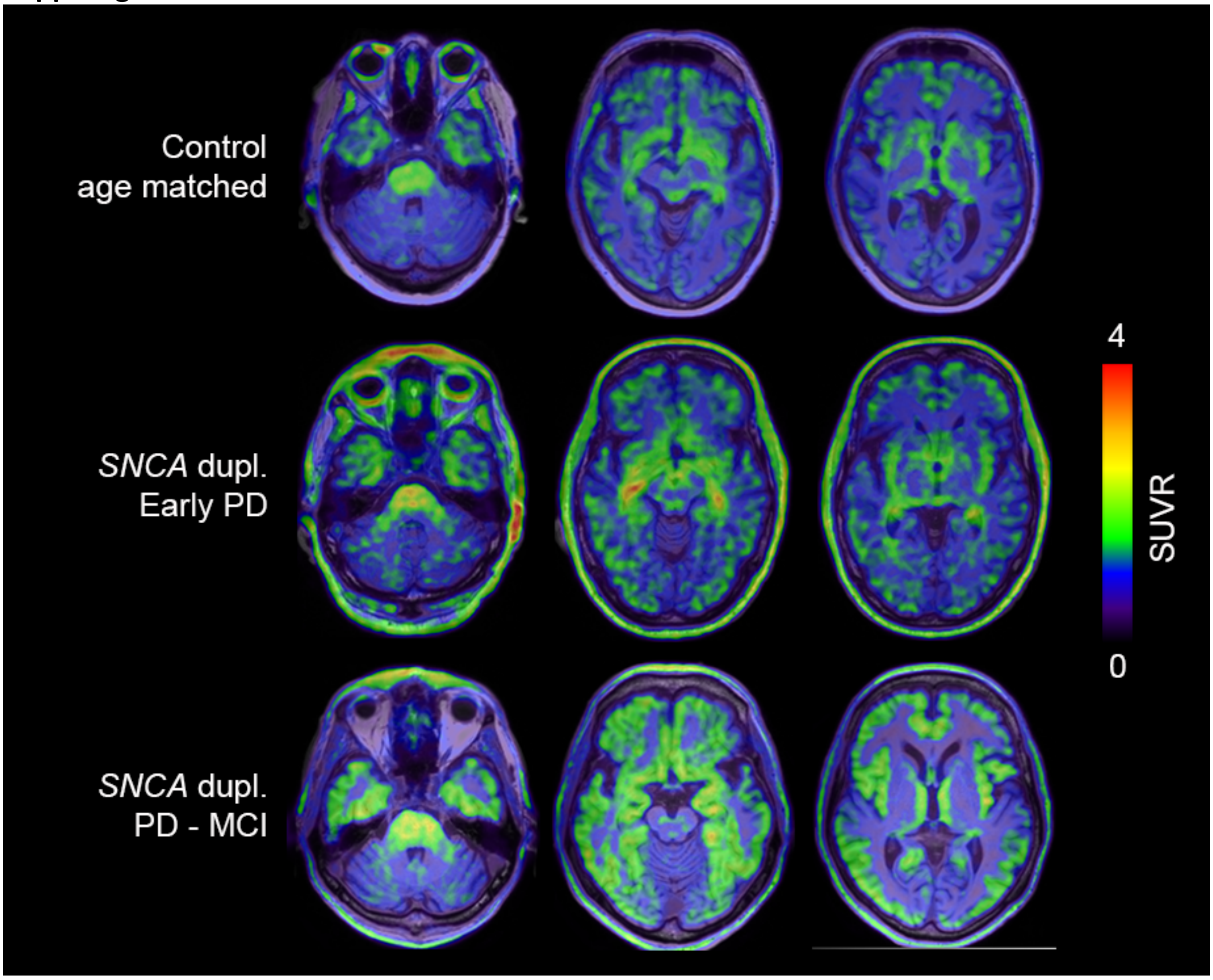

Retention in participants with duplication of the *SNCA* gene.
Transversal [^{18}F]ACI-12589 images at the level of the middle cerebellar peduncles (left row), midbrain (middle row) and the basal ganglia (right row). PD: Parkinson's Disease; SUVR: Standardized uptake value ratio.

Suppl. Figure 15.

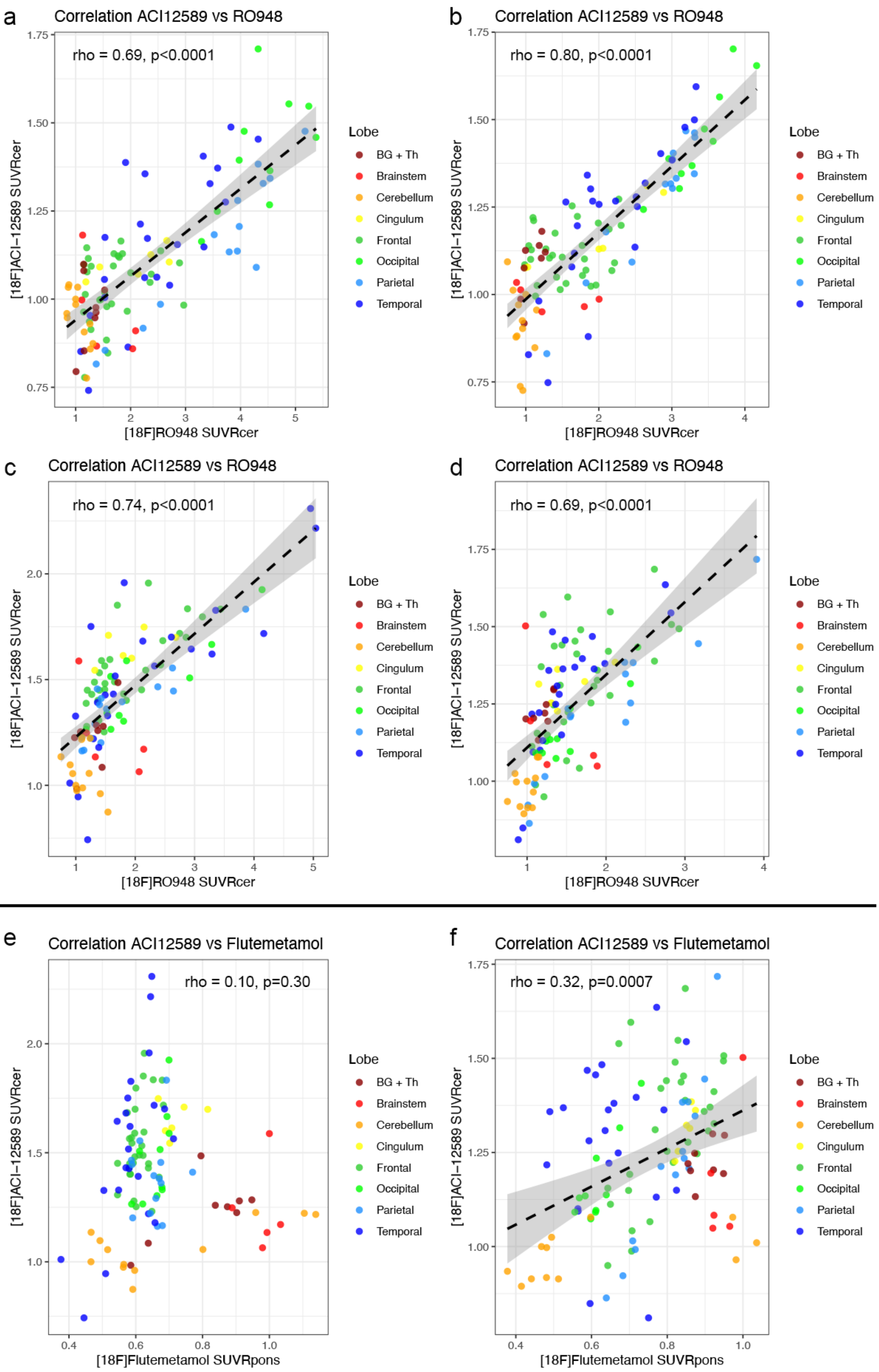

Correlations of [^{18}F]ACI-12589 with [^{18}F]RO948 and [^{18}F]Flutemetamol in participants with AD. Correlations between [^{18}F]ACI-12589 and [^{18}F]RO948 are shown in panels a) to d) for four different participants with AD, correlations between [^{18}F]ACI-12589 and [^{18}F]Flutemetamol in panels e) and f). [^{18}F]Flutemetamol data in e) corresponds to [^{18}F]RO948 data in c) and [^{18}F]Flutemetamol data in f) corresponds to [^{18}F]RO948 data in d). Spearman rho-values and p-values are indicated in the panels. Linear model fits to the data are presented as dashed lines where Spearman correlations were significant. The data points are derived from the automated anatomical labeling atlas from Pmod 3.7 and data from different lobes/brain structures are indicated in different colours. Cerebellum regions included are from the vermis, not included in the cerebellar hemisphere grey matter reference region used for [^{18}F]ACI-12589 and [^{18}F]RO948. Pons was used as a normative region for [^{18}F]Flutemetamol. The error bands (shaded in grey) in the figures represent the 95% C.I. of the dashed linear regression line.
BG + Th: Basal ganglia and thalamus; $SUVR_{cer}$: SUVR with a cerebellar grey matter reference region; $SUVR_{occ}$: SUVR with an occipital cortex reference region; SUVR: Standardized uptake value ratio.

Suppl. Figure 16.

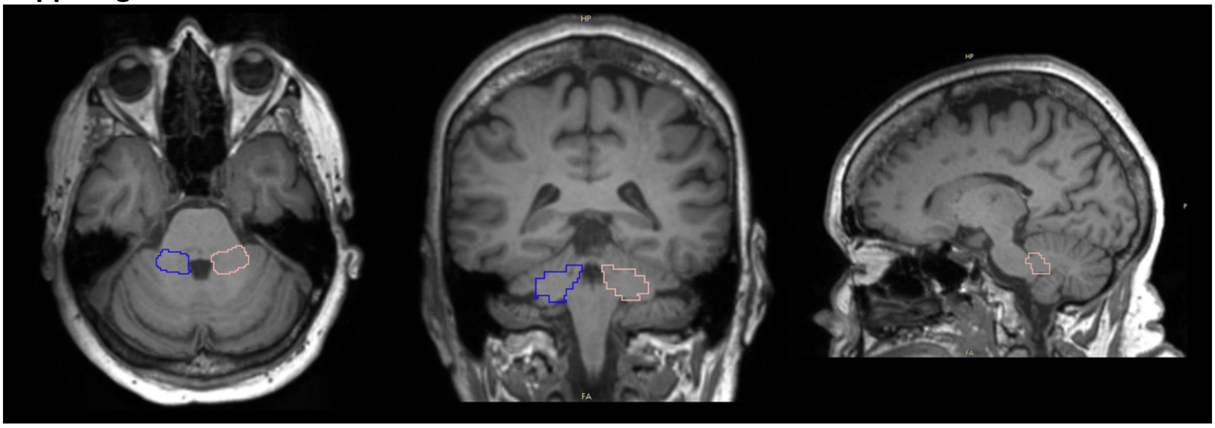

Definition of the middle cerebellar peduncle ROI.

The middle cerebellar peduncle ROI was defined on transversal sections using the MRI data only, but with the MRI transformed into PET space. The anterior limitation of the ROI was the pons, the medial limitation the 4th ventricle and lateral limitation the CSF or the cerebellar cortex in the dorsal parts. The dorsal limitation was defined as a line at the posterior wall of the 4th ventricle.

Suppl. Table 3
Parameters used for analytical HPLC method and chiral HPLC method

	**HPLC method**	**Chiral HPLC method**
Column:	Phenomenex Kinetex® 2.6 µm C18 100 Å, 4,6 x 100 mm, P/N: 00D-4462-E0	Daciel Chiralpak AY-RH, 5 µm, 150 x 4.6 mm, P/N: 47724
Precolumn:	N/A	Daciel Chiralpak AY-RH, 5 µm, 10 x 4 mm, P/N: 47711
Flow:	1.0 ml/min	0.5 ml/min
Column oven:	27 °C	35 °C
UV detection:	254 nm	254 nm
Injection:	10 µL	10 µL
Analysis time:	25 min (analysis and washing step)	30 min
Eluent:	Elution (12 mins):<br>MilliQ water with 0,1% trifluoro acetic acid: Methanol, 82:18.<br>Washing step: 10:90	5 mM ammonium acetate buffer pH 5: Acetonitrile, 45:55

Suppl. Figure 17.

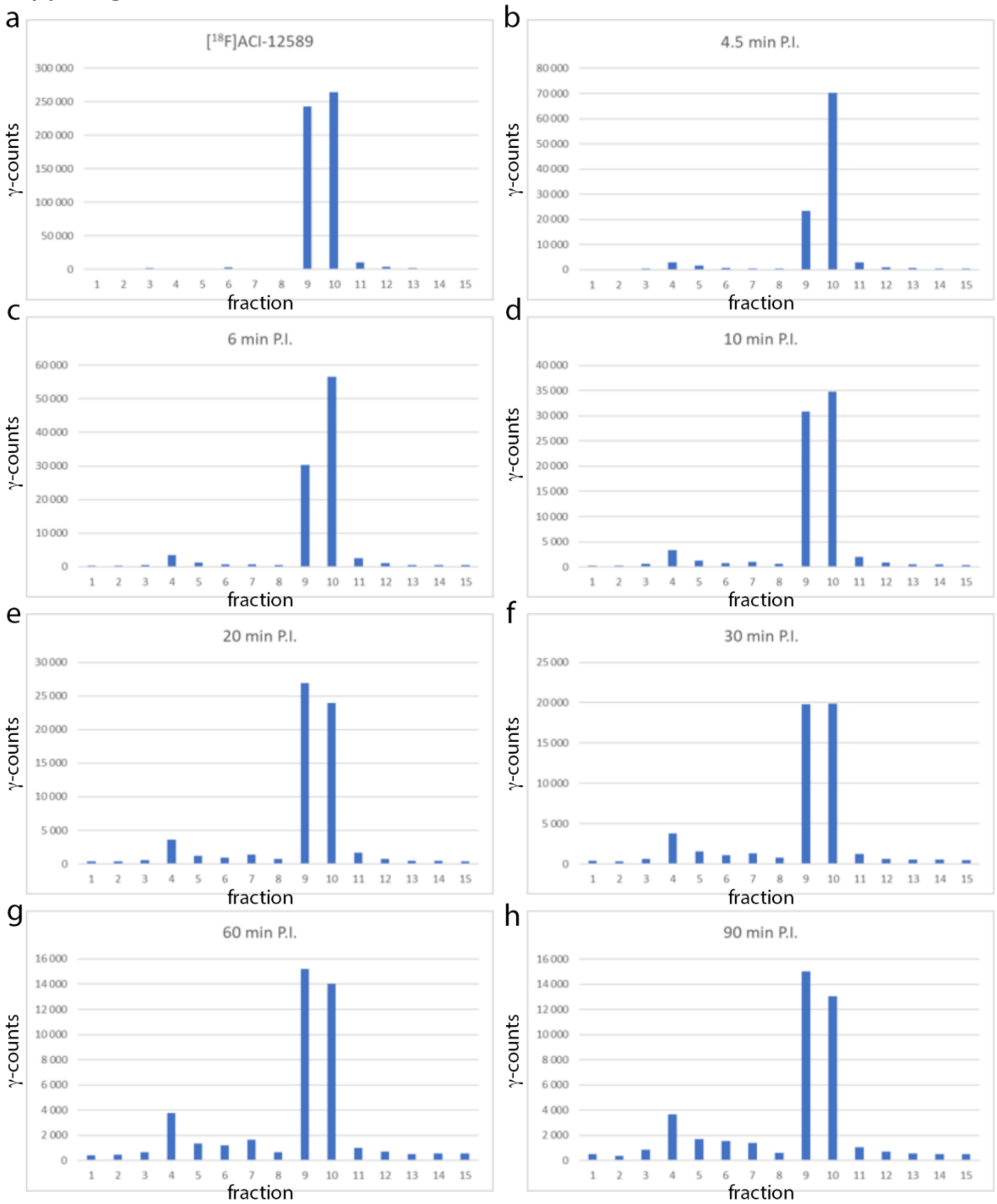

Determination of [^{18}F]ACI-12589 by HPLC in blood samples from one representative patient. [Luna 10µm C18(2) 10 x 250 mm column, flow rate 4 mL/min, 1 minute per fraction, eluent: MeOH:H_2O with 0.2% of triethylamine (60:40)]. a) baseline [^{18}F]ACI-12589; b) 4.5 min P.I.; c) 6 min P.I.; d) 10 min P.I.; e) 20 min P.I.; f) 30 min P.I.; g) 60 min P.I.; h) 90 min P.I.. Fractions 3-8 correspond to free $^{18}F^-$ and metabolites, and fractions to 9-11 intact [^{18}F]ACI-12589. P.I. = post injection.

Supplementary Table 4

Donor code	Diagnosis	Brain region	Age	Sex
PD SNCA	Familial PD, *SNCA* G51D mutation carrier	Frontal cortex	52	Male
PD #1	Parkinson's disease	Amygdala	83	Male
PD #2	Parkinson's disease	Amygdala	58	Female
PD #3	Parkinson's disease	Amygdala	75	Male
PD #4	Parkinson's disease	Amygdala	77	Female
PDD #1	Parkinson's disease with dementia	Amygdala	80	Male
PDD #2	Parkinson's disease with dementia	Amygdala	80	Female
PDD #3	Parkinson's disease with dementia	Amygdala	76	Male
MSA #1	Multiple system atrophy	Cerebellum	70	Male
MSA #2	Multiple system atrophy	Caudate	54	Male
MSA #3	Multiple system atrophy	Cerebellum	62	Female
MSA #4	Multiple system atrophy (Olivopontocerebellar atrophy)	Cerebellum	58	Female
LBV	Lewy body variant of Alzheimer’s disease	Amygdala	83	Male
DLB	Dementia with Lewy Bodies	Cingulate		
AD	Alzheimer’s disease	Frontal cortex, Entorhinal cortex, Amygdala	91	Female
PSP	Progressive Supranuclear Palsy	Basal ganglia	57	Male
NDC #1	Non-diseased control	Middle frontal gyrus	60	Female
NDC #2	Non-diseased control	Amygdala	53	Female
NDC #3	Non-diseased control	Cerebellum	55	Male
NDC #4	Non-diseased control	Amygdala	69	Female
NDC #5	Non-diseased control	Frontal cortex	84	Male
NDC #6	Non-diseased control	Frontal cortex	86	Female

Demographics of the donors for the fresh frozen tissue sections used in the autoradiography studies with [^{18}F]ACI-12589 and [^{3}H]ACI-12589 and high resolution autoradiography studies with [^{3}H]ACI-12589.

Supplementary Table 5
Neuropathological characterization provided by brain banks:

Donor code	Brain region	Age	Brain Bank	Braak and Braak Tau	Braak LB
PD SNCA	Frontal cortex	52	QSBB	β-Amyloid	wide spread LB pathology
MSA #1	Cerebellum	70	QSBB	I	wide spread LB pathology*
NDC #5	Frontal cortex	84	QSBB	I<br>No Tau pathology*	No LB pathology*

* In house characterization by immunofluorescence (pSyn-S129 staining for a-syn and AT8 staining for Tau)

Donor code	Brain region	Age	Brain Bank	Braak	β-Amyloid	Braaklb
PD #1	Amygdala	83	NBB	1	0	6
PD #2	Amygdala	58	NBB	1	0	5
PD #4	Amygdala	77	NBB	0	0	5
PDD #1	Amygdala	80	NBB	1	0	5
PDD #2	Amygdala	80	NBB	4	0	5
PDD #3	Amygdala	76	NBB	1	0	5
MSA #2	Caudate	54	NBB	1	0	6
MSA #3	Cerebellum	62	NBB	1	0	5
LBV	Amygdala	83	NBB	0	0	6
NDC #2	Amygdala	53	NBB	0	0	0
NDC #3	Cerebellum	55	NBB	0	0	0
NDC #4	Amygdala	69	NBB	1	0	0
PSP #2	Globus Pallidus	77	NBB	2	0	3

* In house characterization by immunofluorescence (pSyn-S129 for a-syn aggregates, 4G8 for β-Amyloid, MC1 for Tau)

Donor code	Brain region	Age	Brain Bank	Tau	β-Amyloid	a-syn
NDC #6	Frontal cortex	86	QSBB	+	-	-
MSA #4	Cerebellum	58	NBB	+	-	+
NDC #1	Middle frontal gyrus	60	NBB	-	-	-
PD #3	Amygdala	75	Banner Sun Health Research Institute		-	+

DLB	Cingulate		Transhit Biomarkers	-	+	+
AD	Frontal cortex, Entorhinal cortex, Amygdala	91	Tissue Solutions	+ Braak VI	+	+
PSP	Basal ganglia	57	Tissue Solutions	+	-	+

Supplementary references

1. Bagchi DP*, et al.* Binding of the radioligand SIL23 to alpha-synuclein fibrils in Parkinson disease brain tissue establishes feasibility and screening approaches for developing a Parkinson disease imaging agent. *PLoS One* **8**, e55031 (2013).

2. Tong J*, et al.* Distribution of monoamine oxidase proteins in human brain: implications for brain imaging studies. *J Cereb Blood Flow Metab* **33**, 863-871 (2013).

3. Tonietto M*, et al.* Plasma radiometabolite correction in dynamic PET studies: Insights on the available modeling approaches. *J Cereb Blood Flow Metab* **36**, 326-339 (2016).
