# Supplementary material for: The α-synuclein PET tracer [18F] ACI-12589 distinguishes multiple system atrophy from other neurodegenerative diseases
Source: Nat Commun. 2023 Oct 27;14:6750. doi: 10.1038/s41467-023-42305-3 (PMC10611796; doi:10.1038/s41467-023-42305-3)
Supplement: Supplementary file 3 — Reporting Summary [file 41467_2023_42305_MOESM3_ESM.pdf]

nature portfolio

Corresponding author(s): Oskar Hansson
Johannes Streffer

Last updated by author(s): Oct 1, 2023

Reporting Summary

Nature Portfolio wishes to improve the reproducibility of the work that we publish. This form provides structure for consistency and transparency in reporting. For further information on Nature Portfolio policies, see our Editorial Policies and the Editorial Policy Checklist.

Statistics

For all statistical analyses, confirm that the following items are present in the figure legend, table legend, main text, or Methods section.

n/a	Confirmed	
☐	☒	The exact sample size (*n*) for each experimental group/condition, given as a discrete number and unit of measurement
☐	☒	A statement on whether measurements were taken from distinct samples or whether the same sample was measured repeatedly
☐	☒	The statistical test(s) used AND whether they are one- or two-sided<br>*Only common tests should be described solely by name; describe more complex techniques in the Methods section.*
☐	☒	A description of all covariates tested
☐	☒	A description of any assumptions or corrections, such as tests of normality and adjustment for multiple comparisons
☐	☒	A full description of the statistical parameters including central tendency (e.g. means) or other basic estimates (e.g. regression coefficient) AND variation (e.g. standard deviation) or associated estimates of uncertainty (e.g. confidence intervals)
☐	☒	For null hypothesis testing, the test statistic (e.g. *F*, *t*, *r*) with confidence intervals, effect sizes, degrees of freedom and *P* value noted<br>*Give P values as exact values whenever suitable.*
☒	☐	For Bayesian analysis, information on the choice of priors and Markov chain Monte Carlo settings
☒	☐	For hierarchical and complex designs, identification of the appropriate level for tests and full reporting of outcomes
☐	☒	Estimates of effect sizes (e.g. Cohen's *d*, Pearson's *r*), indicating how they were calculated

Our web collection on statistics for biologists contains articles on many of the points above.

Software and code

Policy information about availability of computer code

Data collection	Data for immunofluorescence experiments were acquired using a Panoramic150 Slide Scanner (3DHistech). For [18F]ACI-12589 autoradiography, slides were scanned using a phosphor imaging system (Fujifilm BAS-5000 phosphor imager, Tokyo, Japan). For [3H] ACI-12589 autoradiography, slides were scanned in a real-time autoradiography system (BeaQuant instrument, ai4R). Data for radiobinding experiments in brain homogenates were acquired on a Microbeta2 scintillation counter (PerkinElmer).<br>Clinical PET data was acquired using GE Discovery MI PET/CT scanners or an ECAT EXACT HR+ scanner as indicated. Raw PET data was reconstructed and attenuation corrected to the CT-scan using machine software into 2D image stacks before being analyzed further as specified below.
Data analysis	Data analysis of PET images were performed using Pmod, version 3.7 (PMOD Technologies LLC, Zurich Switzerland), and kinetic analysis using an in house developed pipeline at NRU, Copenhagen University.<br>Statistical analyses were performed using the R programming language (v 4.0.3) or, where indicated in the methods, using Graph Pad Prism v7.0 .<br>Images resulting from autoradiography with [18F]ACI-12589 were analyzed using Multi Gauge 3.2 phosphor imager software (Fujifilm, Tokyo, Japan). For [3H]ACI-12589 autoradiography, quantification of signal was performed by using the Beamage image analysis software (ai4R).

For manuscripts utilizing custom algorithms or software that are central to the research but not yet described in published literature, software must be made available to editors and reviewers. We strongly encourage code deposition in a community repository (e.g. GitHub). See the Nature Portfolio guidelines for submitting code & software for further information.

Data

Policy information about availability of data

All manuscripts must include a data availability statement. This statement should provide the following information, where applicable:

- Accession codes, unique identifiers, or web links for publicly available datasets
- A description of any restrictions on data availability
- For clinical datasets or third party data, please ensure that the statement adheres to our policy

Anonymized data will be shared by request from a qualified academic investigator for the sole purpose of replicating procedures and results presented in the article and if data transfer is in agreement with EU legislation on the general data protection regulation and decisions by the Swedish Ethical Review Authority, which should be regulated in a material transfer agreement. Source data are provided with this paper.
To access data for reproduction of the results presented herein, please contact corresponding authors.

Research involving human participants, their data, or biological material

Policy information about studies with human participants or human data. See also policy information about sex, gender (identity/presentation), and sexual orientation and race, ethnicity and racism.

Reporting on sex and gender	Both males and females were included in the study, but due to the exploratory nature of the study no between sex comparisons were made. Gender identity was not recorded. The same applies for the donors of the human brain samples.
Reporting on race, ethnicity, or other socially relevant groupings	We did not study race or socioeconomic factors in this manuscript. The same applies for the human brain samples.
Population characteristics	Participants had a median age of 66 years (range 40-81, mean 64.7). 30 were males, 12 females. Participants did not have any other neurological diseases except for the inclusion diagnosis. Other (non-neurological) medical conditions was not an exclusion criterion.<br>The donors of the human brain samples had a median age of 70 years (range 52-91, mean 69.7). 11 were males, 10 females. The neuropathological characterization, provided by brain banks or performed in-house is shown in Suppl. Table 6.
Recruitment	Participants were recruited at the Memory Clinic and the Department of Neurology, Skåne University Hospital, Sweden (Sept 2021 – May 2022) and five PD patients and five normal controls were recruited by Invicro, LLC (New Haven, Connecticut, USA). Participants with synucleinopathies (PD, MSA, DLB), controls and other neurodegenerative diseases were approached and asked to participate in the study. We do not foresee any selection or biases due to recruitment that would affect the outcome of the study.<br>The human brain samples were acquired from the Netherlands Brain Bank (NBB; Netherlands Institute for Neuroscience, Amsterdam (open access www.brainbank.nl)), Queen Square Brain Bank (QSBB), Banner Health Institute Brain & Tissue Bank, and commercial providers (Tissue Solutions Ltd., Glasgow, UK).
Ethics oversight	Written informed consent was obtained from all participants prior to entering the study. The study was approved by the Swedish national Human Research Ethics authority.<br>All human brain samples have been collected from donors for or from whom a written informed consent for a brain autopsy and the use of the material and clinical information for research purposes had been obtained.

Note that full information on the approval of the study protocol must also be provided in the manuscript.

Field-specific reporting

Please select the one below that is the best fit for your research. If you are not sure, read the appropriate sections before making your selection.

☒ Life sciences ☐ Behavioural & social sciences ☐ Ecological, evolutionary & environmental sciences

For a reference copy of the document with all sections, see nature.com/documents/nr-reporting-summary-flat.pdf

Life sciences study design

All studies must disclose on these points even when the disclosure is negative.

Sample size	The study was designed as an exploratory study to evaluate the performance of the novel [18F]ACI-12589 radiotracer. Participants were selected as likely to have alfa-synuclein pathology (i.e. Parkinsons Disease, Lewy body dementia, Multiple system atrophy) or less likely to have alfa-synuclein pathology (Alzheimer's Disease, Progressive Supranuclear Palsy and the hereditary ataxias [Friedreich ataxia and SAMD9L mutations]) as well as controls, not expected to exhibit alfa-synuclein pathology. Due to the exploratory nature of the study no power calculation was performed.
Data exclusions	No data was excluded
Replication	Due to the exploratory nature of this first-in-man study no replication of the data outside of this dataset was performed
Randomization	No randomization was performed. All subjects underwent the same investigations. This also applies to the in vitro-experiments tissues from

	different disease conditions were tested in similar settings.
Blinding	The person (RS) delineating the regions of interest (ROI) for the readout of PET results was blinded to the PET signal when setting the ROI boundaries (done using MRI-data only).

Reporting for specific materials, systems and methods

We require information from authors about some types of materials, experimental systems and methods used in many studies. Here, indicate whether each material, system or method listed is relevant to your study. If you are not sure if a list item applies to your research, read the appropriate section before selecting a response.

Materials & experimental systems

n/a	Involved in the study	
[ ]	[x]	Antibodies
[x]	[ ]	Eukaryotic cell lines
[x]	[ ]	Palaeontology and archaeology
[x]	[ ]	Animals and other organisms
[ ]	[x]	Clinical data
[x]	[ ]	Dual use research of concern
[x]	[ ]	Plants

Methods

n/a	Involved in the study	
[x]	[ ]	ChIP-seq
[x]	[ ]	Flow cytometry
[ ]	[x]	MRI-based neuroimaging

Antibodies

Antibodies used

Antibodies used:
1) Phosphoserine 129 alpha-synuclein (alpha-synuclein-pS129, 1:500, Abcam 51253)
2) The conformation-dependent anti-Tau antibody (MC1, 1:200, kindly provided by Peter Davies, Northwell, USA)
3) anti-pTDP-43 pS409/410 (Biolegend, 829901, 1:500)
4) antibody against MAO-B (Thermofisher, PA5-28338, 1:500).
Secondary antibodies:
5) AlexaFluor647-labelled goat-anti-rabbit antibody (Abcam, ab150079, 1:500)
6) AlexaFluor647-labelled goat-anti-mouse antibody (Jackson ImmunoResearch, 115-605-166, 1:500)
7) AlexaFluor633-labeled goat-anti-rat antibody (Invitrogen, A-21094)

Validation

Information concerning the antibodies used can be found at:
1) https://www.abcam.com/products/primary-antibodies/alpha-synuclein-phospho-s129-antibody-ep1536y-ab51253.html
2) https://www.alzforum.org/antibodies/tau-mc1 and doi: 10.1002/(sici)1097-4547(19970415)48:2<128::aid-jnr5>3.0.co;2-e.
3) https://www.biolegend.com/de-at/products/purified-anti-tdp43-phospho-ser409-410-antibody-11573
4) https://www.thermofisher.com/antibody/product/Monoamine-Oxidase-B-Antibody-Polyclonal/PA5-28338
5) https://www.abcam.com/products/secondary-antibodies/goat-rabbit-igg-hl-alexa-fluor-647-ab150079.html
6) https://www.jacksonimmuno.com/catalog/products/115-605-166
7) https://www.thermofisher.com/antibody/product/Goat-anti-Rat-IgG-H-L-Cross-Adsorbed-Secondary-Antibody-Polyclonal/A-21094

Clinical data

Policy information about clinical studies
All manuscripts should comply with the ICMJEguidelines for publication of clinical research and a completedCONSORT checklist must be included with all submissions.

Clinical trial registration	The study is not an interventional clinical study.
Study protocol	The study is not an interventional clinical study. The study procedures are detailed in the methods section of the manuscript
Data collection	Thirteen MSA, three PD – two of which carried a duplication in the alpha-synuclein (SNCA) gene, two DLB, three PSP, five AD, two with Friedreich Ataxia and one with a mutation in the SAMD9L gene, as well as three normal control participants were recruited at the Memory Clinic and the Department of Neurology, Skåne University Hospital, Sweden (Sept 2021 – May 2022) whereas five PD patients and five normal controls were recruited by Invicro, LLC (New Haven, Connecticut, USA).
Outcomes	The outcome was changes in [18F]ACI-12589 retention in cortical and subcortical ROIs in patients with different synucleinopathies (PD, DLB or MSA) in comparison to controls or patients with other neurodegenerative disorders, such as AD, PSP or hereditary ataxias.

Magnetic resonance imaging

Experimental design

Design type	Structural T1-mprage MR images were acquired for determining brain structure for definition of cortical and subcortical regions of interest (ROIs). The structural MRI was coregistered to the PET image and ROI data was transferred to PET-space for analysis
Design specifications	Participants underwent one 3T MRI with a T1 mprage sequence.
Behavioral performance measures	N/A

Acquisition

Imaging type(s)	Structural T1-mprage
Field strength	3T
Sequence & imaging parameters	MPRAGE (magnetization-prepared, 3D, rapid gradient-echo) anatomical images were acquired with the following acquisition parameters: inversion time = 1100 ms; flip angle = 9°; echo time = 2.54 ms; echo spacing = 7.3 ms; repetition time = 1900 ms; receiver bandwidth = 220 Hz/pixel, and voxel size = 1×1 × 1 mm, scan time = 5:15 min. FOV: 256x256x256
Area of acquisition	whole brain

Diffusion MRI ☐ Used ☒ Not used

Preprocessing

Preprocessing software	Images were reoriented, normalized and coregistered to PET images using Pmod version 3.7 (PMOD Technologies LLC, Zurich Switzerland)
Normalization	Images were reoriented, normalized and coregistered to PET images using Pmod version 3.7 (PMOD Technologies LLC, Zurich Switzerland)
Normalization template	Normalization was performed using Probability Maps Transformation in Pmod version 3.7 (PMOD Technologies LLC, Zurich Switzerland)
Noise and artifact removal	No noise or artifact removal was used
Volume censoring	No censoring was used.

Statistical modeling & inference

Model type and settings	N/A
Effect(s) tested	N/A

Specify type of analysis: ☐ Whole brain ☒ ROI-based ☐ Both

Anatomical location(s)	The AAL atlas (as implemented in Pmod 3.7) was used as an initial template. All subcortical ROIs were then checked manually (using the MRI data only) and adjusted not to include CSF and not to include automatically included erroneous voxels in other areas. The middle cerebellar peduncles were delineated as specified in the supplementary information.
Statistic type for inference (See Eklund et al. 2016)	N/A
Correction	N/A

Models & analysis

n/a	Involved in the study
☒	☐ Functional and/or effective connectivity
☒	☐ Graph analysis
☒	☐ Multivariate modeling or predictive analysis
